# Blood leukocyte transcriptional modules and differentially expressed genes associated with disease severity and age in COVID-19 patients

**DOI:** 10.1038/s41598-023-28227-6

**Published:** 2023-01-17

**Authors:** Silvia Y. Bando, Fernanda B. Bertonha, Sandra E. Vieira, Danielle B. L. de Oliveira, Vanessa N. Chalup, Edison L. Durigon, Patricia Palmeira, Ana Cristina P. Curi, Caroline S. Faria, Leila Antonangelo, Gerhard da P. Lauterbach, Fabiane A. Regalio, Roberto M. Cesar Jr, Carlos A. Moreira-Filho

**Affiliations:** 1 Department of Pediatrics, Faculdade de Medicina da Universidade de São Paulo, São Paulo, SP 05403-900 Brazil; 2 Instituto Israelita de Ensino e Pesquisa Albert Einstein, Hospital Israelita Albert Einstein, São Paulo, SP 01310-200 Brazil; 3 Department of Microbiology, Laboratory of Clinical and Molecular Virology, Institute of Biomedical Sciences, Universidade de São Paulo, São Paulo, SP 05508-040 Brazil; 4 Laboratório de Investigação Médica (LIM03), Hospital das Clínicas, Faculdade de Medicina da Universidade de São Paulo, São Paulo, SP 01246-903 Brazil; 5 Department of Internal Medicine, Faculdade de Medicina da Universidade de São Paulo, São Paulo, SP 01246-903 Brazil; 6 Divisão de Anestesia, Hospital das Clínicas da Faculdade de Medicina da Universidade de São Paulo, São Paulo, SP 01246-903 Brazil; 7 Department of Computer Science, Instituto de Matemática e Estatística da Universidade de São Paulo, São Paulo, SP 05508-040 Brazil

**Keywords:** Pathogenesis, SARS-CoV-2

## Abstract

Since the molecular mechanisms determining COVID-19 severity are not yet well understood, there is a demand for biomarkers derived from comparative transcriptome analyses of mild and severe cases, combined with patients’ clinico-demographic and laboratory data. Here the transcriptomic response of human leukocytes to SARS-CoV-2 infection was investigated by focusing on the differences between mild and severe cases and between age subgroups (younger and older adults). Three transcriptional modules correlated with these traits were functionally characterized, as well as 23 differentially expressed genes (DEGs) associated to disease severity. One module, correlated with severe cases and older patients, had an overrepresentation of genes involved in innate immune response and in neutrophil activation, whereas two other modules, correlated with disease severity and younger patients, harbored genes involved in the innate immune response to viral infections, and in the regulation of this response. This transcriptomic mechanism could be related to the better outcome observed in younger COVID-19 patients. The DEGs, all hyper-expressed in the group of severe cases, were mostly involved in neutrophil activation and in the p53 pathway, therefore related to inflammation and lymphopenia. These biomarkers may be useful for getting a better stratification of risk factors in COVID-19.

## Introduction

The molecular mechanisms determining COVID-19 severity in different age groups are not yet well understood. The study of blood transcriptomic differences between mild and severe cases of the disease seems to be a promising strategy for discovering these mechanisms, moreover when the information derived from the genomic analyses is combined with clinico-demographic and laboratory data obtained from patients’ cohorts, in a systems biology approach^1,2^. Additionally, the obtention of gene expression profiles for severe and mild cases, and for patients’ age groups as well, together with the identification and functional characterization of differentially expressed genes, may contribute to a better stratification of risk factors. It may also provide a thorough understanding of COVID-19 pathogenesis, helping the choice of adequate therapies^3–5^ and, through the deciphering of host response transcriptional complexity, the discovery of new therapeutic targets^5^.

Consequently, we decided to investigate the transcriptional basis of the differences between severe and mild cases of COVID-19 through a comparative study of the transcriptional responses of human leukocytes to SARS-CoV-2 infection in different age groups. We adopted a Weighted Gene Co-expression Network Analysis (WGCNA) approach^6^ for identifying transcriptional modules associated with the traits of interest (severity, age) and, subsequently, we conducted differential gene expression and enrichment analyses for discovering transcriptional biomarkers, as further described in the following paragraphs.

## Materials and methods

### Ethics statement

This study was approved by the Research Ethics Committee of the Hospital das Clínicas da Faculdade de Medicina da Universidade de São Paulo (HC-FMUSP) under number 4.001.109. A written informed consent was obtained from all participants or from their legal guardians. All methods were performed in accordance with the relevant guidelines and regulations.

### Characteristics of participants

The participants included 121 SARS-CoV-2 PCR positive subjects recruited between May and August 2020, before the emergence of the first variant of concern in Brazil, that occurred in November 2020^7^ (Supplementary Table S1). All these patients were unvaccinated against SARS-CoV-2. They were divided into two groups—Mild or Severe—according to the severity of illness categories described in the NIH COVID-19 Guidelines [COVID-19 Treatment Guidelines Panel. Coronavirus Disease 2019 (COVID-19) Treatment Guidelines. National Institutes of Health. Available at https://www.covid19treatmentguidelines.nih.gov/, accessed on 12/07/21]. The Severe group comprised 58 COVID-19 hospitalized patients requiring oxygen therapy that have been tested positive for SARS-CoV-2 by RT-qPCR and were admitted at the HC-FMUSP. The Mild group comprised 63 individuals recruited as outpatients who presented at least one of the following symptoms: fever, coryza, dyspnea, anosmia or hyposmia, ageusia or hypogeusia, wheezing in the chest, diarrhea, vomiting, body pain, headache, sore throat, or chills (Table 1). They were tested by nasopharyngeal swab RT-qPCR and found to be positive for SARS-CoV-2 and negative for other 15 respiratory viruses. After 14 days the clinical evolution of signs or symptoms of all these individuals was checked and the diagnosis of oligosymptomatic COVID-19 was confirmed.

**Table 1:** Clinical and demographic characteristics of the COVID-19 hospitalized (Severe group) and oligosymptomatic (Mild group) patients.

	Severe	Mild	*p*-value
Number of subjects	58	63	
Male	35 (60%)	25 (40%)	**0.012**
Median age	50 yrs	34 yrs	**0.002**
Male (range)	49 yrs (1 mo–81.4 yrs)	35 yrs (8 mo–79.8 yrs)	0.055
Female (range)	51 yrs (0 mo–75 yrs)	32 yrs (2.3–73.2 yrs)	**0.020**
0–40 yrs			
Male	13	15	
Female	6	26	0.022*
> 40 yrs			
Male	22	10	
Female	17	12	0.411*
Group 0–9 yrs (median)	0	5	**0.015**
Group 10–40 yrs (median)	36	29.5	**0.041**
Group > 40 yrs (median)	56.5	53	0.083
Race (no. subjects)			
White	37	37	0.302**
Mixed (Pardo)	21	11	0.140**
Asian	0	2	0.496**
Symptoms (no. subjects)	Yes, No; U	Yes, No; U	
Cough	47; 11	45; 11; 5	0.325
Fever	42; 16	30; 28; 5	**0.011**
Coryza	12; 46	46; 12	**0.000**
Dyspnoea	42; 12	17; 41; 5	**0.000**
Wheezing in the chest	17; 40; 1	2; 56; 5	**0.000**
Anosmia/hyposmia	30; 23; 5	32; 24; 7	0.478
Ageusia/hypogeusia	27; 26; 5	32; 24; 7	0.260
Headache	25; 28; 5	8; 50; 5	**0.000**
Sore throat	34; 19; 5	31; 27; 5	0.128
Diarrhea	25; 33	17; 41; 5	0.062
Vomiting	15; 41; 2	3; 55; 5	**0.000**
Body pain	21; 31; 6	14; 43; 6	0.047
Chills	33; 23; 2	0; 44; 19	**0.027**
Therapy (no. subjects)			
Oxygen	49; 4; 5	NA	
Antibiotics	42; 11; 5	9; 49; 5	0.000*
Antiviral drugs	12; 41; 5	1; 57; 5	0.001*
Corticosteroids	19; 34; 5	3; 55; 5	0.000*
Hospital stay/age groups	Median hospital stay (days)		
1 mo–9 yrs	7		0.311 (vs 10–40 yrs)
			0.045 (vs > 41 yrs)
10–40 yrs	6		**0.033** (vs > 41 yrs)
> 41 yrs	9		
Blood sample collection after symptoms onset (range)			
	Median 13 days (2–69 d)	Median 10 days (4–27 d)	< **0.0001**

*U* unknown, *NA* not applicable, *mo* months, *yrs* years, *d* days.

Thirteen patients in the Mild group opted for not answering the question relative to race.

All statistical analyses were performed using Mann Whitney test, except for the analyses indicated by * or ** where Chi-square or Fisher exact tests were used.

In bold, significant *p*-values < 0.05.

### Factor analysis of mixed data (FAMD)

FAMD is a principal component method for analyzing a data set containing both numerical and categorical variables^8^. To perform this analysis, the Severe and Mild groups were divided into three age ranges: (i) 0 months to nine years; (ii) 10–40 years; (iii) > 41 years. Numerical data was obtained from Complete Blood Count (CBC) and age, while the qualitative levels of CBC variables (low, normal, or high) according to age and sex were included as categorical variables together with sex, symptoms, and other variables of potential clinical relevance (Table 1). This analysis excluded missing values and comprised 64 variables and 92 individuals (Supplementary Fig. S1). FAMD was performed using the package FactoMineR version 2.7^9^ in R-studio environment (version 1.2.5033)^10^.

### Sample collection, processing, and analysis

Whole blood samples were collected from hospitalized patients (median of 13 days after first symptoms) and from oligosymptomatic individuals (median of 10 days after first symptoms). All samples were collected in EDTA-containing tubes. Each blood sample was divided for complete blood count analysis, white blood cell and plasma separation. White blood cells were used for RNA extraction. Neutrophil-to-lymphocyte ratio (NLR), platelets-to-lymphocyte ratio (PLR), systemic immune-inflammation index (SII = (P × N)/L), and neutrophil-to-platelet ratio (NPR) were calculated as these hemogram-derived ratios correlate with inflammation and COVID-19 severity^11^.

Nasopharyngeal and oropharyngeal samples were collected through a swab and/or a mucus specimen trap and kept under refrigeration (4 °C) up to 24 h until cryogenic cooling and storage at − 70 °C for further molecular diagnosis of SARS-CoV-2 and/or other respiratory viruses.

#### Plasma cytokine concentrations

Cytokines (IL-2, IL-4, IL-6, IL-10, IL-17, IFN-gamma, and TNF-alpha) were measured in plasma samples by flow cytometry using a BD™ Cytometric Bead Array (CBA) Human Th1/Th2/Th17 Cytokine Kit (BD Biosciences, San Jose, CA) according to the manufacturer’s instructions, and the concentrations were expressed in pg/mL. Plasmatic IFN-alpha and IFN-beta were evaluated by ELISA (IFN beta and IFN alpha Duoset ELISA, R&D Systems Inc., Minneapolis, MN) and the concentrations were expressed in pg/mL. Cytokine data were censored if they were below the detection limit of the instrument^12^.

#### Detection of respiratory viruses by RT-qPCR

Nasopharyngeal and oropharyngeal samples from oligosymptomatic individuals were obtained for RNA extraction using the NucliSens easyMag® platform fully automated (BioMerieux, Lyon, France), according to the manufacturer’s instructions. RT-qPCR was employed for detecting the genetic material of SARS-CoV-2 and of 15 other respiratory viruses: influenza A virus (Inf A), influenza B virus (Inf B), seasonal coronaviruses (CoV-NL63, -229E, -HKU1, and -OC43), enterovirus (EV), parainfluenza viruses (PIV-1, -2, -3 and -4), human metapneumovirus (HMPV), rhinovirus (RV), respiratory syncytial virus (RSV), and adenovirus (AdV). A panel of validated in-house singleplex qPCR assays developed at the Centers for Disease Control and Prevention (CDC, Atlanta, GA, USA) was used according to Corman et al.^13^ for SARS-CoV-2, and according to Sakthivel et al.^14^ for the other viruses, using TaqMan™ assays (Applied Biosystems). For the reactions (25 μL of final volume), the AgPath-ID™ One-Step RT-PCR Kit (Applied Biosystems) was used, and the amplification step was carried out on the ABI 7500 instrument (Applied Biosystems) under the following cycling conditions: 45 °C for 10 min (1 cycle); 95 °C for 10 min (1 cycle); and 95 °C for 15 s, followed by 55 °C for 1 min (45 cycles).

#### RNA extraction

A total of 0.5–1.5 mL of whole blood was used for white blood cells (WBC) RNA extraction. WBC were immediately separated by centrifugation using EL buffer (QIAamp RNA Blood Mini kit, Qiagen, Hilden, Germany). After cell separation, the WBC were collected, preserved in RNA*later* (Qiagen) and stored at 10 °C until RNA extraction. WBC were lysed with RLT buffer, and the total RNA was extracted using QIAamp^®^ RNA Blood Mini kit (Qiagen). RNA purity analysis and quantification were performed using the NanoVue spectrophotometer (GE Heathcare Life Sciences, Marlborough, MA). RNA quality was assessed on the Agilent BioAnalyzer 2100 (Agilent, Santa Clara, USA). All RNA samples were stored at − 80 °C until used in hybridization experiments.

### Microarray hybridization

A total of 23 RNA samples were used for gene expression analysis and grouped as Severe (n = 11) or Mild (n = 12) according to the patient’s characteristics (Supplementary Table S2). These two groups showed no significant differences regarding age, sex, and time of whole blood collection after the first day of COVID-19 symptoms. The Severe group was further divided into subgroups A and B, where A included the samples from adolescents and younger adults (age ranging from 11 to 38 years; n = 5) and B included the samples from older adults (age ranging from 41 to 62 years; n = 6). Similarly, the Mild group was divided in subgroups C and D, where C included the samples from adolescents and younger adults (age ranging from 10 to 37 years; n = 7) and D included the samples from older adults (age ranging from 41 to 64 years; n = 5). The patients between zero and nine years (Severe and Mild groups) were not included due to insufficient RNA quality.

To determine gene expression profiles, 4 × 44 K DNA microarrays (Whole Human Genome Microarray Kit, Agilent Technologies, cat no. G4845A) were used. The procedures for hybridization using the fluorescent dye Cy3 followed the manufacturer’s protocols (One-Color Microarray-Based Gene Expression Analysis—Quick Amp Labeling). The images were captured by the reader Agilent Bundle according to the parameters recommended for bioarrays and extracted by Agilent Feature Extraction software version 11.5.1.1 (https://www.agilent.com/). Spots with two or more flags (low intensity, saturation, controls, etc.) were considered as NA, that is, without valid expression value. All microarray raw data have been deposited in GEO public database (http://www.ncbi.nlm.nih.gov/geo), a MIAME compliant database, under accession number GSE193022.

### Gene expression analysis

An in-house algorithm in R environment (version 3.6.2)^10^ was used for excluding transcripts presenting one or more missing values (NAs) per group and for converting gene expression values to log base 2. Through this procedure we obtained two gene expression data matrices: (i) one for gene co-expression network (GCN) analysis, and (ii) another for differential gene expression (DEG) analysis. The GCN matrix had 6375 Gene Ontology (GO) annotated genes after excluding all NAs. The DEG matrix had 15,248 GO annotated genes, including NAs and with a minimum of four valid gene expression values per group. Boxplot analysis was used for outlier detection (Supplementary Fig. S2). Data normalization for both data matrices was performed using limma package version 3.9^15^ in R environment (version 3.6.2)^10^. The differential gene expression analyses were conducted in three comparisons separately: (i) Severe *vs.* Mild (ii) A *vs.* B; (iii) C *vs.* D.

### Weighted gene co-expression network analysis (WGCNA)

The network was constructed using the WGCNA package^16^ (version 1.69-81; https://horvath.genetics.ucla.edu/html/CoexpressionNetwork/Rpackages/WGCNA/) in R version 3.6.2 environment^10^. No outliers were detected in the analysis of gene expression data matrix. Pearson’s correlation coefficient was used for obtaining gene co-expression similarity measures and for the subsequent construction of an adjacency matrix using soft power and topological overlap matrix (TOM). Soft thresholding process transforms the correlation matrix to mimic the scale-free topology. TOM is used to filter weak connections during network construction. Module identification is based on TOM and on average linkage hierarchical clustering. The soft power β = 20 (R^2^ = 0.9160) was chosen using the scale-free topology criterion (Supplementary Fig. S3). Finally, Dynamic Tree Cut algorithm^6^ was used for dendrogram’s branch selection. The module eigengene (ME) is defined as the first principal component of a given module, which can be considered a representative of the gene expression profiles in a module. Module Membership (MM), also known as eigengene-based connectivity (*k*ME), is defined as the correlation of each gene expression profile with the module eigengene of a given module.

#### Module-trait association

Module-trait association analysis was accomplished using the WGCNA package (version 1.69-81)^16^ in R environment (version 3.6.2)^10^. For this analysis we considered as specific traits: severity group, age subgroup, sex, race, comorbidities, therapy, clinical laboratory characteristics, hospital stay, and blood collection time point after first symptoms onset. Only traits present in three or more patients were considered in the module-trait association analysis. Subsequently, the gene significance (GS), i.e., a value for the correlation between specific traits and gene expression profiles^16^ was obtained. The mean GS value for a particular module is considered as a measure of module significance (MS). Modules presenting a significant *p*-value (*p* < 0.05) and a positive trait correlation were selected for functional analysis.

#### Node categorization

All genes belonging to a given module were designated here as ME genes (for module eigengene gene). The modules significantly correlated to specific traits (age group, clinical, and laboratorial data) were further evaluated for identifying relevant hub genes, i.e., the highly connected genes, here termed high hierarchy (HH) genes, that hold the transcriptional network together and are also associated to specific cellular processes or link different biological processes^17^. Thus, connectivity measures were used for the hierarchical categorization of hub genes, considering connectivity values related to the network (overall connectivity) and to the module (intramodular connectivity for each gene based on its Pearson’s correlation with all other genes in the module).

Intramodular node connectivity was calculated considering: (i) *k*Total, the whole network connectivity of each gene; (ii) *k*Within, gene connections with other genes in the same module^16^. Genes presenting high *k*Total and *k*Within are classified as high hubs (Hhubs), genes presenting high *k*Total but low *k*Within are called eHubs, and genes presenting high *k*Within but low *k*Total are the iHubs. The iHubs connect most of the genes in a transcriptional module, whereas the eHubs connect different transcriptional modules, and the Hhubs hold the transcriptional modules and the network together^18^. The top 10 genes presenting the highest *k*Total and/or *k*Within values were selected as HH genes. All gene values were plotted in a *k*Total (x-axis) *vs. k*Within (y-axis) graph. Additionally, the expression profile was assessed for the selected hubs through GS values, i.e., the GS of the nth gene is the correlation measure between the nth gene expression and the specific trait. Positive or negative GS values mean that the nth gene is hyper- or hypo-expressed for the specific trait. Only GS values with *p* < 0.01 were considered significant for the trait.

### Enrichment analyses

WGCNA modular gene set enrichment analyses for Gene Ontology Biological Process (GO BP), KEGG pathways, and Reactome pathways were accomplished by using the Enrichr online web-based tool^19^. The terms presenting adjusted *p*-value < 0.05 for the modules or *p*-value < 0.05 for the hub genes, were considered significantly enriched. The same enrichment analysis strategy was applied for all hub genes and DE genes.

### Statistical analysis

Significance analysis for microarray (SAM) using MeV software (version 4.9.0) was used for differential gene expression analyses. The relative expression of the differentially expressed genes (DEGs) was normalized with the endogenous reference gene *GUSB* for statistical analysis (Supplementary Table S3). The t-test was used for statistical analyses of differentially expressed genes. Mann Whitney, Chi-square, and Fisher exact tests were used for statistical analysis of the clinic-demographical data. All statistical analyses were performed in GraphPad Prism (version 8). The FAMD analysis was performed as described in “Factor analysis of mixed data (FAMD)” section.

### Gene co-expression network (GCN)

The GCNs were constructed by using Pearson’s correlation. Data analysis and visualization were accomplished through Cytoscape^20^ version 3.9.0.

## Results

### Patient demographic and clinical characteristics

The clinical and demographic characteristics of the severe (hospitalized) and mild (oligosymptomatic) COVID-19 patients are shown in Table 1. The median age for the Severe and Mild groups was significantly different: 50 years and 34 years, respectively. There was a predominance of males in the Severe group as compared to the Mild group. There were more young male patients (0–40 years of age) in the Severe group. Significant lower median hospital stay was observed for young patients (10–40 years of age) as compared with elderly patients (Table 1). In the Severe group more patients presented fever, dyspnea, wheezing in the chest, and vomiting, while more oligosymptomatic individuals presented coryza and headache (Table 1). A significantly higher number of patients in the Severe group presented at least one comorbidity. In decreasing order of frequency: systemic arterial hypertension (SAH), obesity, diabetes mellitus (DM), chronic heart conditions, chronic lung diseases, and chronic kidney disease (Supplementary Fig. S4).

The hemogram results were interpreted as normal, high, or low, based on reference values for age and sex (Supplementary Table S4). The comparative analysis between the Severe and Mild groups showed that more individuals in the Severe group presented high values for segmented neutrophils and low values for erythrocytes, hematocrit, hemoglobin, lymphocytes, and immature neutrophils (Supplementary Fig. S5). Severe patients had significantly elevated values for all hemogram-derived ratios when compared to Mild (i.e., oligosymptomatic) patients (Supplementary Table S5), and higher values were found for PLR and SII parameters in the subgroups B and D (patients over 41 years of age), thus confirming the positive correlation of elevated hemogram-derived ratios with inflammation and severe COVID-19^10^. Additionally, the individuals in the Severe group expressed higher levels of IL-6 and IL-10 when compared with the Mild group. The cytokines IL-4, IL-2, and IFN-γ, were also elevated in these individuals (Supplementary Fig. S6).

**Figure 1:**
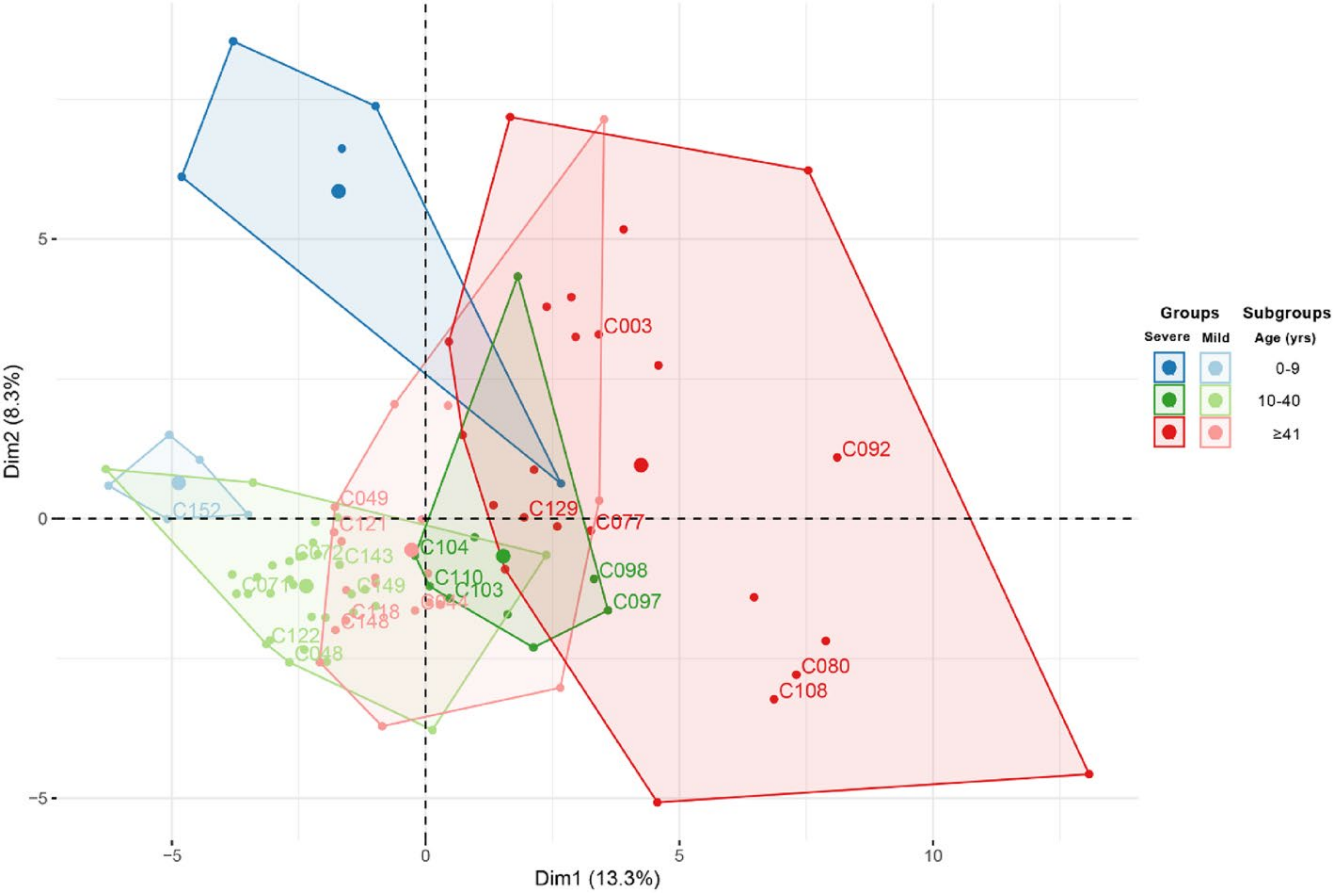
Exploratory multivariate analysis. Factor analysis of mixed data (FAMD) with grouping variables identified four dissimilar groups: the two main groups (Severe and Mild), and the two age-related subgroups (10–40 years old; 41–85 years old).

FAMD analysis for clinical characteristics yielded six dimensional clusters for patients aged 0–9, 10–40 and over 40 years, in the Severe and Mild groups (Fig. 1). All patients selected for transcriptomics studies are contained in four dimensional clusters: 10–40 and over 40 years for both Severe and Mild groups.

### WGCNA and module-trait correlation analysis

The normalized gene expression data of 6375 GO annotated genes were used for network construction and module identification by WGCNA. Nine transcriptional modules were identified. Module sizes ranged from 147 genes in the magenta module to 891 genes in the turquoise module (Supplementary Fig. S7). The hierarchical clustering dendrogram of module-eigengenes revealed two meta-modules. In the meta-module I, which encompasses the blue, red, turquoise, magenta, pink, black, and yellow modules, the transcriptional modules are correlated with severe phenotypes, whereas the in the meta-module II, that includes the brown and green modules, the correlation is with mild phenotypes (Supplementary Fig. S8a). The module-trait correlation analysis identified three transcriptional modules with at least one significant correlation (*p* < 0.05) with disease severity (Severe; Mild) or with the A and B age subgroups (Supplementary Figs. S7, S8b). The yellow module is highly (r = 0.70) and positively correlated with the Severe group and with high level of segmented neutrophils (r = 0.72), and weakly (r = 0.46) correlated with subgroup B (old and severe patients) and with low level of lymphocytes (r = 0.51). The magenta module is positively correlated with the Severe group (r = 0.44), subgroup A (young and severe patients, r = 0.47), and high level of segmented neutrophils (r = 0.46). It is also positively correlated with male sex in the Severe group (r = 0.47) and with white race (r = 0.55), and negatively correlated with DM in the Severe group (r =  − 0.62). The black module is positively correlated with subgroup A (r = 0.48) and with high level of segmented neutrophils (r = 0.48), and negatively correlated with DM in the Severe group (r =  − 0.63).

We also performed a functional characterization of these three transcriptional modules and identified their high hierarchy (HH) genes (Supplementary Fig. S9). Transcriptional modules often represent biological processes that can be phenotype-specific^18^. The functional enrichment among the genes within a module is widely used for disclosing its biological meaning^18^.

#### Yellow module

**Figure 2:**
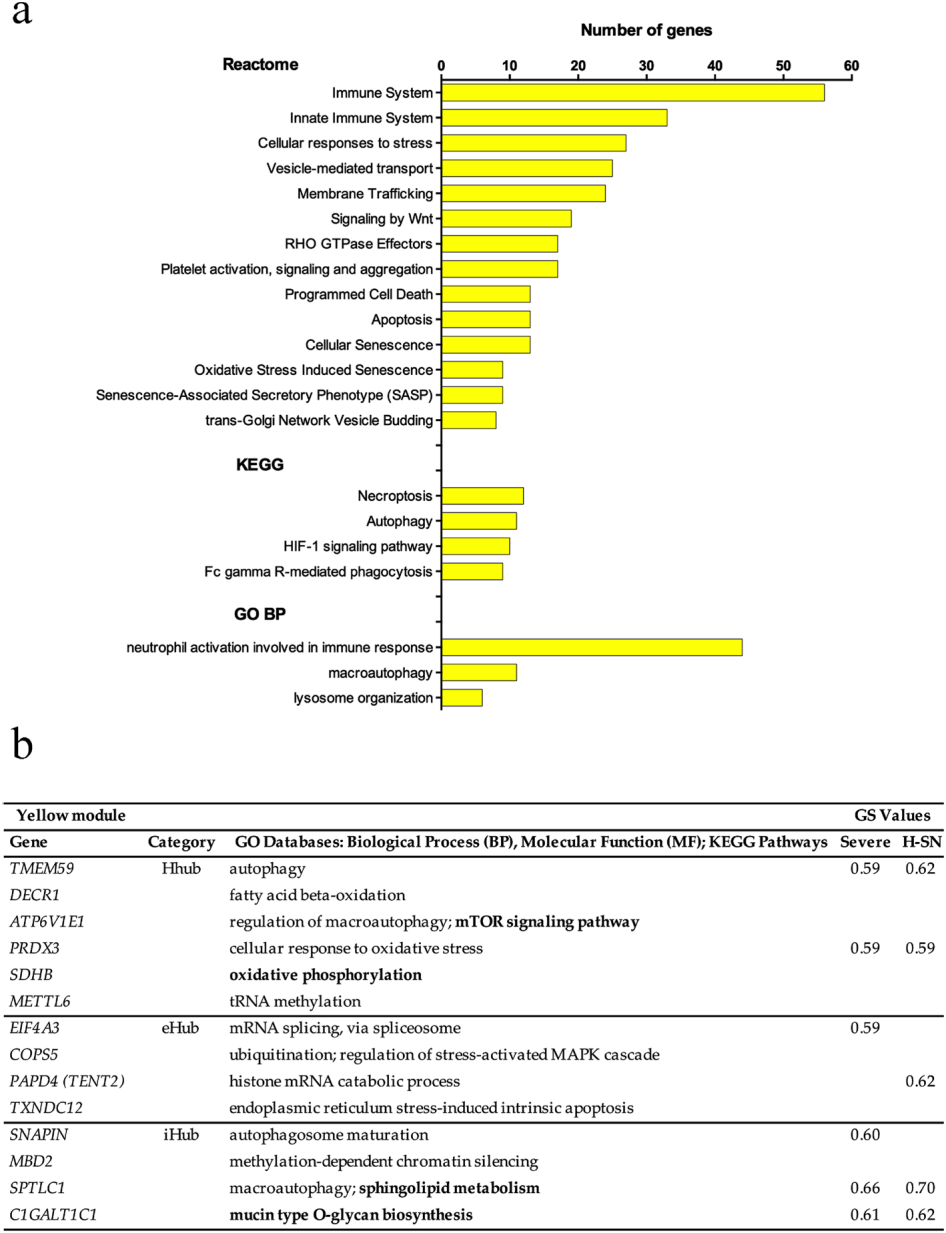
Enrichment analysis and high hierarchy genes (HH genes) for the yellow module. (**a**) Histogram of enriched Reactome, KEGG pathways, and GO BP terms. The terms with adjusted *p* < 0.05 were considered significant. (**b**) HH genes (Hhubs, iHubs, and eHubs) of this module, which is significantly and positively correlated with Severe group, subgroup B, H-SN, and L-Lymphs traits. Each HH gene is identified by its hierarchical categorization, GO biological process or molecular function, and KEGG Pathways-related terms (in bold letters). Only positive (i.e., hyper-expressed) or negative (i.e, hypo-expressed) significant GS values for the specific trait (*p* < 0.01) are shown. *H-SN* high percentage of segmented neutrophils in the hemogram, *GS* gene Significance.

The enrichment analyses for this module (Fig. 2a, Supplementary Table S6) showed that the most represented cellular processes and functional pathways mainly reflect the inflammatory and innate immune responses against SARS-CoV-2^21^. Two terms are worth mentioning: (i) in the GO BP analysis, the most represented category is related to neutrophil activation (44 genes), what is quite expectable due to the role of neutrophils in restricting viral replication and diffusion^22^; (ii) in the KEGG analysis, the genes of the HIF-1 signaling pathway, that are hyper-expressed in the peripheral blood mononuclear cells (PBMC) of COVID-19 patients^23^ and aggravate inflammatory responses^24^.

The yellow module harbors 14 HH genes (Fig. 2b). The Hhubs *TMEM59*^25^ and *ATP6V1E1*^26^, and the iHubs *SPTLC1*^27^ and *SNAPIN*^28^ are autophagy-related genes, the latter being critical for autophagosome maturation in macrophages. Two Hhubs—*PRDX3*^29^ and *SDHB*^30^—are involved in the protection against oxidative stress. The eHub *TXNDC12* (alias *ERP16*) takes part in the cellular defense against prolonged ER stress^31^. A decrease in ER-stress was shown to occur in a stage-specific manner during neutrophil and macrophage differentiation^32^. The iHub *C1GALT1C1* codes for a molecular chaperone (Cosmc) that plays a crucial role TRAIL-induced apoptosis^33^. A well-balanced IFN/TRAIL response is necessary for overcoming viral infections^34^. Finally, it is important to mention that in this module seven HH genes also have high GS values. Four of these genes—*TMEM59*, *PRDX3*, *SPTLC1*, and *C1GALT1C1*—are positively correlated with the Severe group and with high level of segmented neutrophils. Two other genes—*EIF4A3*, a modulator of the non-sense mediated mRNA decay pathway^35^ and *SNAPIN*—are positively correlated with the Severe group. *TENT2* (alias *PAPD4*), a gene involved in miRNA processing^36^, is positively correlated with high level of segmented neutrophils. Taken together, the gene enrichment analyses and the functional characterization of the HH genes for the yellow module fairly agree with the positive correlation of this module with COVID-19 severe cases, with subgroup B (aged and severe patients), and with high number of segmented neutrophils and lymphopenia (i.e., elevated NLR and SII; Supplementary Table S5), two hallmarks of COVID-19 severity^37^.

#### Magenta module

**Figure 3:**
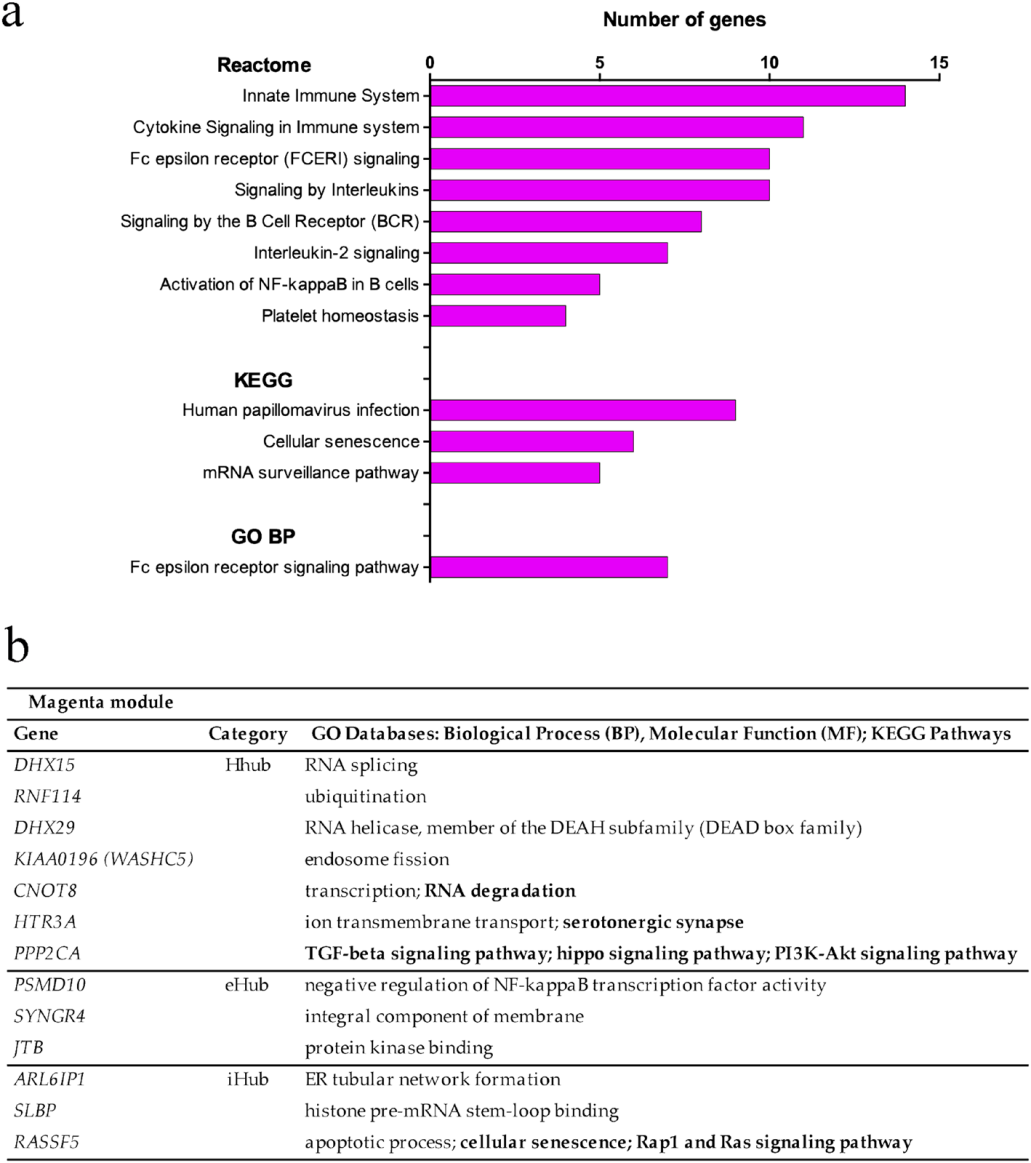
Enrichment analysis and high hierarchy genes (HH genes) for the magenta module. (**a**) Histogram of enriched Reactome, KEGG pathways, and GO BP terms. The terms with adjusted *p* < 0.05 were considered significant. (**b**) HH genes (Hhubs, iHubs, and eHubs) of this module, which is significantly and positively correlated with Severe group, subgroup A, and with H-SN traits. Each HH gene is identified by its hierarchical categorization, GO biological process or molecular function, and KEGG Pathways-related terms (in bold letters). This module did not present significant Gene Significance (GS) values for any specific trait (*p* < 0.01).

The enrichment analyses revealed a predominance of pathways related with innate and adaptive immune responses and inflammation (Fig. 3a, Supplementary Table S7). Moreover, the Fc epsilon receptor signaling pathway indicates the involvement of basophils and, indeed, it was recently shown that these cells play an active role in the immunity against SARS-CoV-2^38^.

The magenta module has 13 HH genes (Fig. 3b) and most of them act on the modulation of the innate and adaptive antiviral immune responses. The Hhub *PPP2CA* codifies protein phosphatase 2A, an important regulator of inflammatory signaling^39^. The Hhub *RNF114* codes for a RING-type zinc-finger protein that modulates NF-κB activity and T-cell activation^40^. The Hhub *HTR3A* encodes the subunit A of the 5-HT3 receptor of serotonin, found in monocytes, B and T cells and probably involved in their recruitment to sites of inflammation^41^. Decreased serum levels of serotonin were found in severe cases of COVID-19, whereas in the mild cases the serotonin levels were close to those found in control individuals^42^. The Hhubs *DHX15*^43^ and *DHX29*^44^ code for two DEAH-box helicases that are key sensors for antiviral defense against RNA virus infection. The Hhub *WASHC5* codes for strumpellin, a protein involved in endosomal fission^45^ that was shown to interact directly with SARS-CoV-2^46^. The Hhub *CNOT8* encodes the CNOT8 catalytic subunit of the CCR4-NOT complex, which is required for selective mRNA degradation (mRNA surveillance) and for preventing excessive inflammatory responses^47^. The eHub *PSMD10* codes for gankyrin and promotes autophagy^48^, a crucial innate immune response against infection. The eHub *SYNGR4* codes for the integral membrane component synaptogyrin and promotes endosome recycling^49^ that is essential for cytokine release by neutrophils^50^. The eHub *JTB* (alias *PAR*) acts on the regulation of mitochondrial function^51^ and the low expression of mtDNA in the immune system cells of COVID-19 patients was recently reported^52^. Lastly, there are three iHubs: *SLBP*, which encodes a TNF-induced stem-loop binding protein that regulates histone metabolism, inflammation, and viral replication^53^*. RASSF5* (alias *NORE1A*), an apoptosis inducer and senescence effector^54^; and *ARL6IP1*, that codes for an ER-shaping protein/ involved in the fine control of ER organization^55^.

#### Black module

**Figure 4:**
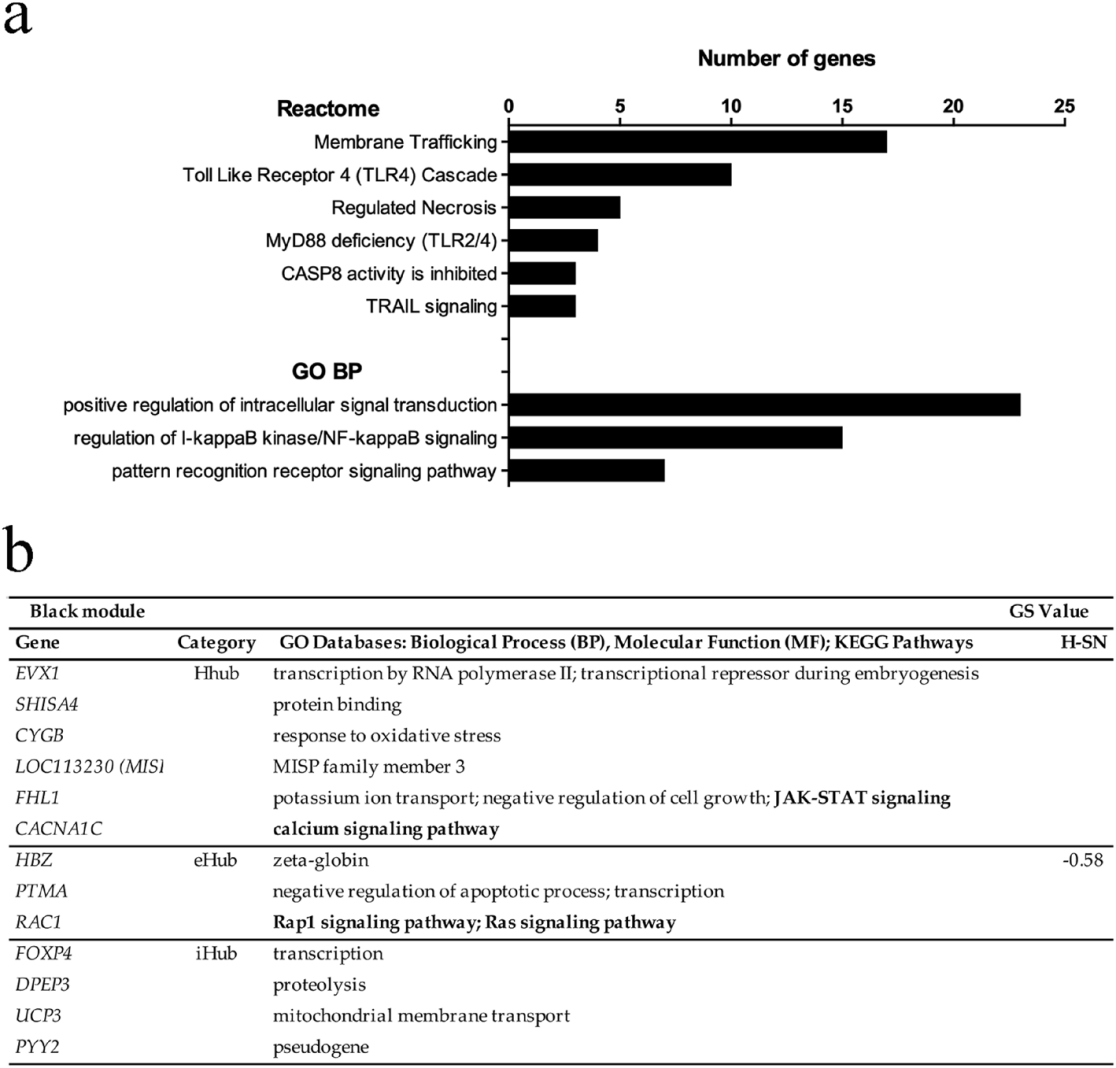
Enrichment analysis and high hierarchy genes (HH genes) for the black module. (**a**) Histogram of enriched Reactome and GO BP terms. The terms with adjusted *p* < 0.05 were considered significant. (**b**) HH genes (Hhubs, iHubs, and eHubs) of this module, which is significantly and positively correlated with subgroup A and with H-SN traits. Each HH gene is identified by its hierarchical categorization, GO biological process or molecular function, and KEGG Pathways-related terms (in bold letters). Only positive (i.e., hyper-expressed) or negative (i.e, hypo-expressed) significant GS values for the specific trait (*p* < 0.01) are shown. *H-SN* high per-centage of segmented neutrophils in the hemogram, *GS* gene significance.

The enriched pathways in the black module (Fig. 4a, Supplementary Table S8) reflect some of the main mechanisms involved in the innate immune and inflammatory responses to SARS-CoV-2. Many pathways, such as TLR/4, MyD88/TLR2, CASP8, TRAIL signaling, and necrosis (Fig. 4a) are mechanistically related and take part in the recognition of viral proteins, inflammatory responses, and cell death^56^. Interestingly, the increased expression of TLRs and MyD88 were found to be positively correlated with COVID-19 severity^57^. Noteworthy, the GO BP analysis shows enrichment for I-kappaB Kinase/NF-kappaB signaling pathway, a master regulator of inflammatory and immune responses^58^.

This module has 13 HH genes (Fig. 4b) of which five are directly related to immune response regulation. The Hhub *CACNA1C* codes for the alpha-1 subunit of CaV1.2 calcium channel and is involved in the regulation of human Th2-lymphocyte functions^59^. The eHub *RAC1* codifies a Rho GTPase that triggers NF-κB activation^60^. The eHub *PTMA* encodes prothymosin-alpha, an alarmin involved in cell proliferation, apoptosis, and immune regulation and whose expression in CD8 T memory stem cells is increased in COVID-19^61^. The iHub *FOXP4* codifies a transcription factor required for T cell recall response to pathogens^62^. The iHub *UCP3* codes for an uncoupling protein (UC) involved in the maintenance of Th17/Treg cell balance^63^, which is skewed towards Th17 in COVID-19^64^.

The other HH genes in the black module are involved in different biological functions, some of them possibly related to modulatory mechanisms acting on inflammatory and immune responses. The Hhub *CGYB* codes for cytoglobin, a protein that protects cells against oxidative stress^65^. The Hhub *FHL1* encodes a four-and-a-half-LIM-domain protein involved in gene transcription regulation, cytoarchitecture, cell proliferation, and signal transduction^66^. Interestingly, FHL1 inhibits the vascular endothelial growth factor (VEGF) expression^67^ and elevated serum levels of VEGF were positively correlated with disease severity in COVID-19^68^. The Hhub *MISP3* codes for a protein involved in spindle orientation and mitotic progression^69^. The Hhub *EVX1* encodes a transcriptional repressor^70^ and the Hhub *SHISA4* codes for a transmembrane adaptor protein^71^. The iHub *DPEP3* encodes dipeptidase 3, involved in the hydrolytic metabolism of dipeptides, and the iHub *PYY2* is a pseudogene coding for peptide YY2. Finally, the eHub *HBZ* codes for zeta hemoglobin and HBZ expression may be interpreted as evidence for circulating erythroid precursors in hospitalized (requiring oxygen) patients^72^, being negatively correlated with hemoglobin levels. *HBZ* has a negative GS value for the H-SN trait (Fig. 4b).

### Differential gene expression analyses

Two comparative gene expression analyses (SAM) were conducted: (i) Severe (n = 11) *vs.* Mild (n = 12) groups; (ii) A (n = 5) *vs.* B (n = 6) subgroups. In the Severe *vs.* Mild comparison 50 differentially expressed genes (DEGs) were found, of which 49 were hyper-expressed in the Severe group, with fold-changes ranging between 8.8 and 2.0. The A *vs.* B comparison yielded only two DEGs. Subsequently, a normalized expression analysis (using *GUSB* as endogenous reference) showed that these two genes did not differ significantly between the subgroups A and B.

The enrichment analysis for the DEG set of the Severe group showed that these genes are mainly related with neutrophil activation, innate immune response, and inflammation (Supplementary Fig. S10, Table S9).

A normalized gene expression analysis (using *GUSB* as an endogenous reference gene) was then performed for the DEGs firstly selected according to KEGG and GO BP enrichment analyses. It was found that 23 genes in the Severe group were significantly (*p* < 0.005) hyper-expressed (Table 2). Noteworthy, 17 out of the 23 genes hyper-expressed in the Severe group had been previously reported to be hyper-expressed in the PBMC from COVID-19 patients according to the COVID-19 Related Gene Sets, a database in the Enrichr webtool library^19^ (Table 2). It is also important to mention that 13 out of the 23 genes hyper-expressed in the Severe group are related with neutrophil activation.

**Table 2:** DEGs selected as potential biomarkers for the Severe group and for the subgroup C.

Comparison	DEG	Gene expression		Database for enrichment analysis
		Fold-change	Relative (*p*-value)*	GO BP or KEGG (in italic)
Severe × Mild	***LTF***	8.53	0.0001	Neutrophil activation involved in immune response; negative regulation of apoptotic process
	HP**	6.95	0.0002	Neutrophil activation involved in immune response; positive regulation of cell death
	***CEACAM8***	6.16	0.0009	Neutrophil activation involved in immune response
	HPR**	5.64	0.0003	Acute inflammatory response; positive regulation of cell death
	***LCN2***	5.02	0.0013	Neutrophil activation involved in immune response
	***ARG1***	4.98	0.0009	Neutrophil activation involved in immune response
	GYG1**	4.06	0.0002	Neutrophil activation involved in immune response
	***MPO***	4.03	0.0011	Neutrophil activation involved in immune response; negative regulation of apoptotic process
	*ORM1*	3.98	0.0008	Neutrophil activation involved in immune response; platelet degranulation
	*TXNDC5*	3.66	0.0020	Neutrophil activation involved in immune response; negative regulation of apoptotic process
	ORM2**	3.56	0.0006	Neutrophil activation involved in immune response; platelet degranulation
	***CAMP***	3.27	0.0006	Neutrophil activation involved in immune response
	RRM2**	2.95	0.0002	p53 signaling pathway
	GGH**	2.86	0.0004	Neutrophil activation involved in immune response
	*SELP*	2.71	< 0.0001	Platelet degranulation
	CCNB1**	2.57	0.0004	**p53 signaling pathway; cellular senescence**
	BMP6**	2.43	0.0001	**Hippo signaling pathway**
	BIRC5**	2.37	< 0.0001	**Hippo signaling pathway;** negative regulation of apoptotic process
	SNCA**	2.33	0.0004	Positive regulation of cell death; negative regulation of oxidoreductase activity
	YWHAH**	2.25	0.0005	**Hippo signaling pathway**
	CCNB2**	2.23	< 0.0001	**p53 signaling pathway; cellular senescence**
	STOM**	2.22	0.0004	Neutrophil activation involved in immune response
	***CDK1***	2.14	< 0.0001	**p53 signaling pathway; cellular senescence;** negative regulation of apoptotic process

Genes in bold are hyper-expressed in the PBMC from COVID-19 patients (COVID-19 Related Gene Sets/Enrichr database).

*t-test for relative expression of the DEGs normalized with endogenous reference gene *GUSB*.

**Newly identified as a potential COVID-19 transcriptional biomarker.

#### DEGs as transcriptional biomarkers

The significant fold-change values and the biological functions of all DEGs found for the Severe group (Table 2, Supplementary Figs. S11, S12) clearly show their potentiality as transcriptional biomarkers. The ascribed roles of these genes—of which 13 are newly identified biomarkers (Table 2)—in the immune response to COVID-19 are addressed in the following paragraphs.

Among the seven genes in the GO BP category “neutrophil activation involved in immune response” (Supplementary Fig. S11), three were already identified as COVID-19 biomarkers: *CEACAM8* (alias *CD66B*), that codes for a neutrophil cell-adhesion protein and is highly expressed in patients with severe COVID-19^73^. *ARG1*, coding for arginase 1 and up-regulated in severe cases of COVID-19^74^; and *LCN2*, which encodes lipocalin 2, a marker of neutrophil activation that was classified by machine learning algorithm as one the most potent discriminators of critical illness in COVID-19^75^. One DEG in this category, *CAMP*, codify for the antimicrobial molecule cathelicidin (LL37), a modulator of TLR activation and inflammation that has been proposed as a potential candidate for COVID-19 prevention and treatment^76^. The remaining three DEGs are related to neutrophil immune functions and metabolism: *STOM*, that codes for stomatin, a membrane protein associated with azurophilic granules^77^; *GGH*, that encodes gamma-glutamyl hydrolase, a lysosomal enzyme involved in the immune response of neutrophils^78^; and *GYGY* that codifies glycogenin, an enzyme involved in the synthesis of glycogen^79^.

In the GO BP category “neutrophil activation and positive regulation of cell death” (Supplementary Fig. S6) there are two DEGs. One is *HP* that codes for haptoglobin, a hemoglobin-binding plasma protein stored and released by neutrophils in response to activation^80^. Haptoglobin plasmatic levels were shown to correlate with disease severity in COVID-19, being reduced in critical patients^81^ and elevated in COVID-19 children^82^. The other DEG, *HPR*, codes for a haptoglobin-related protein that binds hemoglobin was shown to be elevated in the sera of children with bacterial and viral pneumonia^83^. In the GO category “positive regulation of cell death and negative regulation of oxidoreductase activity” there is only one DEG, *SCNA*, which codes for alpha-synuclein, a neuropeptide expressed in brain and also in mononuclear blood cells^84,85^ that was shown to exhibit potent antiviral activity and capacity for signaling the immune system by attracting neutrophils and macrophages and activating dendritic cells^86,87^.

There are three DEGs in the GO BP category “neutrophil activation and negative regulation of apoptosis” (Supplementary Fig. S11). One is *LTF*, which codifies for lactoferrin, a relevant player in innate immunity with antiviral effects against SARS-CoV-2 and a wide range of viral species^88^. Serum levels of lactoferrin were found to be elevated in severe cases of COVID-19^89^. The other DEG is *MPO*, a gene that codes for myeloperoxidase, a leukocyte-derived enzyme whose plasmatic levels are elevated in mild to severe cases of COVID-19 and down-regulated in patients with very severe disease^90^. The third gene is *TXND5*, that codes for the thioredoxin domain containing 5 (TXNDC5), an endoplasmic reticulum-resident protein that belongs to the thioredoxin family. The plasmatic levels of this protein are markedly elevated in septic patients, and it has been considered a therapeutic target for attenuating inflammatory responses^91^. Additionally, it was shown that *TXND5* is hyper-expressed in B cells after COVID-19 vaccination, being a marker for seroconversion^92^. Therefore, the three genes are potential candidates for monitoring disease severity in COVID-19.

The GO BP category “platelet degranulation and neutrophil activation” has three DEGs (Supplementary Fig. S11). One of these genes is *SELP*, that codes for P-selectin, a platelet cell-adhesion molecule. Increased levels of P-selectin were found in severe cases of COVID-19, what contributes for a prothrombotic state in these patients^93^. The other two genes in this category are *ORM1* and *ORM*2, and both are involved in the encoding of human orosomucoid protein, a major acute-phase plasma protein^94^. Recently, a proteomic analysis of serum from COVID-19 patients showed a significant down-regulation of the *ORM1* protein^95^.

The KEGG pathway “p53 signaling and cellular senescence” (Supplementary Fig. S12) includes four DEGs. One of these genes is *CDK1* that codes for the cyclin-dependent kinase 1 and is a master regulator of autophagy^96^. *CDK1* was shown to be, through bioinformatics and machine learning approaches, a relevant Hub gene in the WBC transcriptome of COVID-19 patients, with high biomarker and therapeutic target potentials^97^. Other two DEGs in this pathway, *CCNB1* and *CCNB2*, coding respectively for cyclin B1 and cyclin B2, are interactors of *CDK1* and regulate the mammalian cell cycle^98^. The fourth DEG in this pathway, *RRM2*, interacts with *CDK1* and *CNBB1* in the p53 pathway^99^ and was recently identified, through bioinformatic analysis, as a key gene in the infection of human intestines by SARS-CoV-2^100^.

Finally, there are three DEGs in the KEGG “Hippo signaling pathway” (Supplementary Fig. S12). One of these genes is *BIRC5* that codes for survivin, an inhibitor of apoptosis protein. Survivin is indispensable for the homeostasis of the immune system, being required for innate and adaptive immune responses, differentiation of CD4+ and CD8+ memory T-cells, and for B cell maturation^101^. The other two DEGs are *BMP6* and *YWHAH*. The former codes for the bone morphogenetic protein 6, a regulator of vascular homeostasis and angiogenesis^102^ recently identified as an anti-inflammatory cytokine^103^. The latter codes for the 14-3-3η (eta) protein, a phospho-serine/phospho-threonine binding protein that interacts with a wide range of protein targets and participates in multiple cellular biological functions. The 14-3-3η protein is involved in the modulation of antiviral defenses via the RLR signaling pathway. The interaction between 14-3-3η and the melanoma differentiation-associated gene 5 (*MDA5*) accelerates the activation of the MDA5 signaling, thus helping host cells to mount an effective response against RNA viral infections^104^.

### Subnetworks for module genes and DEGs

**Figure 5:**
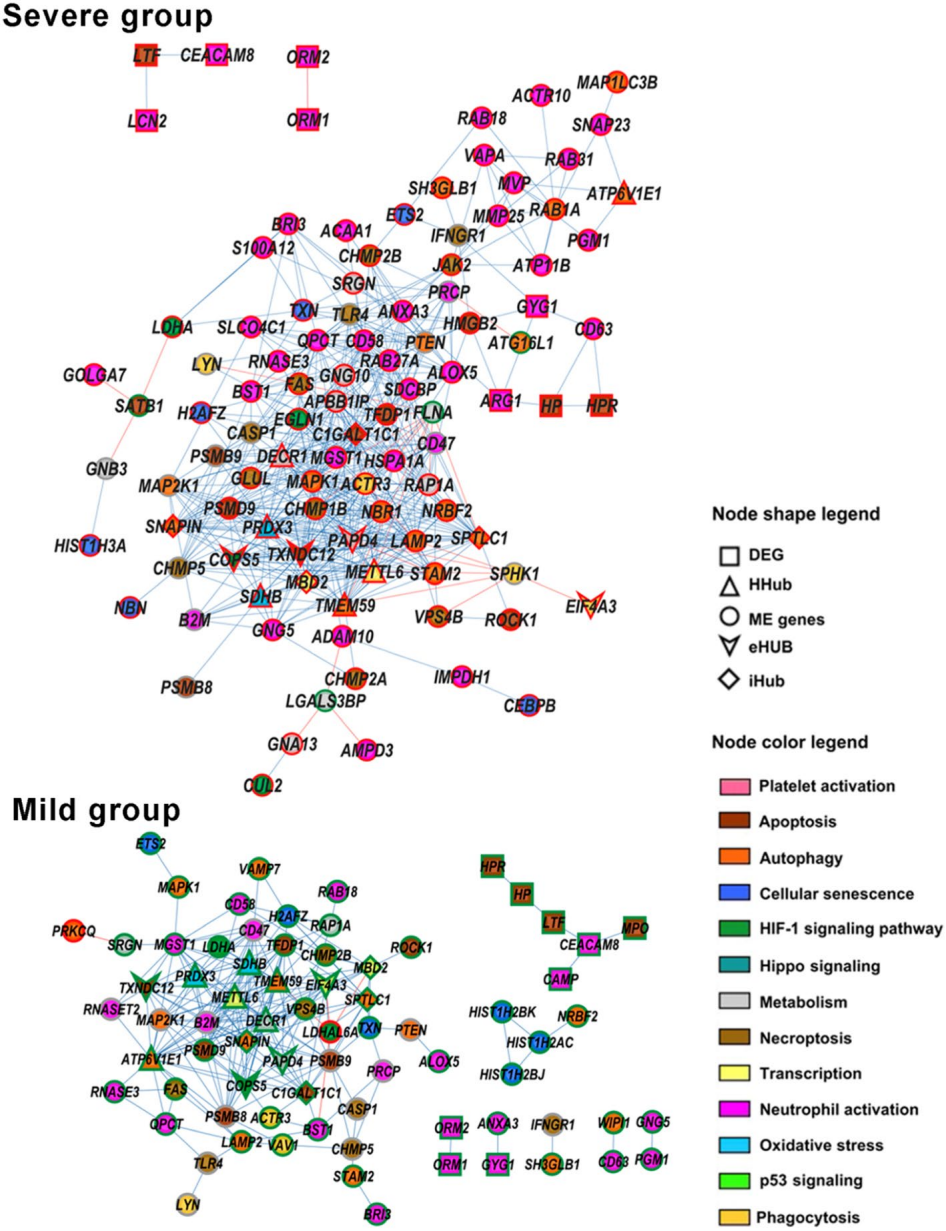
Subnetworks for the yellow module containing ME genes (non-HH module genes), hub genes, and DEGs for the Severe and Mild groups. Red or blue links indicate positive or negative expression correlation values, respectively. Red or green border colors account for hyper-expressed or hypo-expressed genes, respectively.

**Figure 6:**
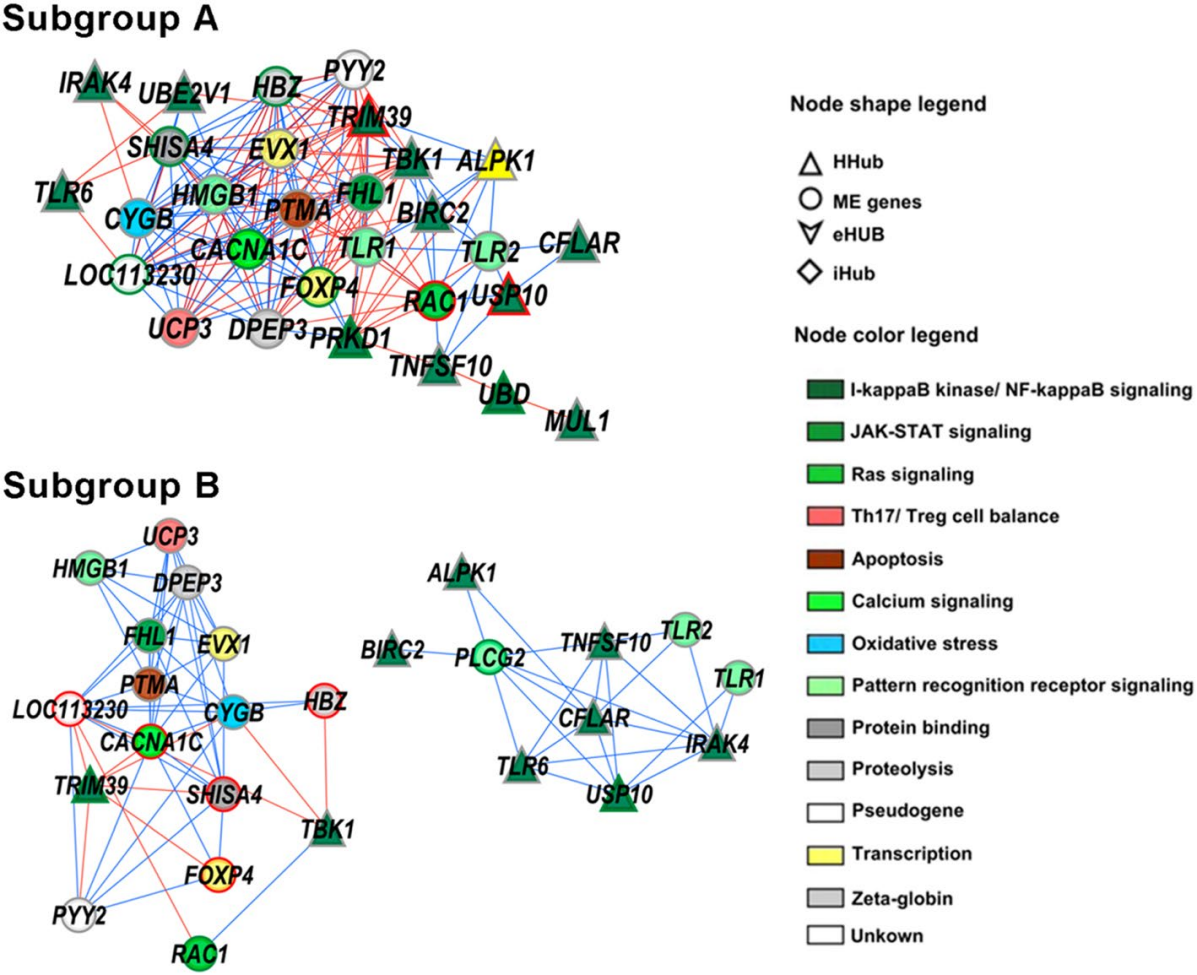
Subnetworks for the black module containing ME genes (non-HH module genes), hubs, and DEGs for the subgroups A and B. Red or blue links indicate positive or negative expression correlation values, respectively. Red or green border colors account for hyper-expressed or hypo-expressed genes, respectively.

Subnetworks were constructed for the yellow and black modules. The subnetwork for the yellow module, positively correlated with the Severe group, was constructed using genes enriched for innate immune response and inflammation processes and pathways. The subnetwork for the black module, positively correlated with subgroup A, was constructed using genes enriched for inflammation, innate immune response, and immune response regulation. All these genes and their respective functions are listed in Supplementary Table S10. The DEGs identified in the Severe *vs.* Mild group comparison were also included in the yellow subnetwork (Supplementary Table S9). Consequently, these subnetworks were constructed with 141 genes for the yellow subnetwork and 33 genes for the black subnetwork. For all subnetworks a gene–gene link cut-off of r >|0.9| was adopted. The yellow subnetwork (Fig. 5) for the Severe group showed a higher value of connectivity (10.6) when compared with the subnetwork obtained for the Mild group (3.2). The subnetworks for the black module (Fig. 6) showed a higher connectivity for subgroup A (11.3) when compared with the subgroup B (6.0). Additionally, the yellow subnetwork contains more interconnected genes in the Severe group (104 genes and 551 links) than in the Mild group (74 genes and 241 links). It is worth to mention that the yellow subnetwork for the Severe group encompasses several genes involved in neutrophil activation (Fig. 5). Similarly, the black subnetwork for subgroup A contains many genes involved in the I-kappaB Kinase/NF-kappaB signaling, an important modulator of inflammatory and immune responses (Fig. 6).

## Discussion

The transcriptomic response of human leukocytes to SARS-CoV-2 infection was investigated by focusing the differences between mild and severe cases and between age subgroups (younger and older adults). The gain of this investigation strategy consists in combining host response transcriptomic data with clinical, demographic, and laboratory information, in a systems biology approach, for studying the immune response between mild and severe COVID-19^1,2,105^. Here, it was possible to functionally characterize the transcriptional modules correlated with age and disease severity, as well as to identify severity-associated DEGs, as discussed below. Still, this study faced two limitations, i.e., the relatively small number of patients included in the transcriptomic study (Table S2), thus impeding a statistical evaluation of the recovery parameter in these patients, and the reduced number of infants presenting severe symptoms, preventing us to investigate the leukocyte transcriptional response to SARS-CoV-2 in that young population.

Initially, it is worth to recall that most of the patients in the Severe group presented fever, dyspnea, wheezing in the chest, chills, and vomiting, while in the Mild group a sizable number of patients solely presented mild symptoms, such as coryza and headache. It is well known that when the response to respiratory viruses is inadequate the infection spreads to lower respiratory tract (LRT)^106^. An early and effective immune response in patients infected with SARS-CoV-2 can limit and eliminate the infection yet in the upper respiratory tract (URT)^107^. Genomic analyses shown that there is no tissue specific genetic adaptation of SARS-CoV-2 to the URT or LRT^108^.

The transcriptional module differences between the Severe and Mild groups reflect and are coherent with the different presentation of symptoms and with COVID-19 severity. The functional enrichment analysis of the yellow module, associated with the Severe group, revealed that this module is related with inflammation and innate immune response, such as neutrophil activation, apoptosis, necroptosis, and HIF-1 signaling pathway. Expectedly, this module contains seven out of its 14 HH genes highly correlated with disease severity and/or high level of segmented neutrophils. Increased neutrophil count, immature circulating neutrophils, and neutrophil activation transcriptomic sig-natures are typically found in severe cases of COVID-19^37^. Interestingly, the HIF-1 pathway is associated with neutrophil activation, prolonged lifetime, and excessive function in COVID-19 severe patients^24,37^.

Furthermore, the subnetwork constructed for the yellow module showed that most of the highly interconnected genes in the Severe group are hyper-expressed, and that many of these genes are involved in neutrophil activation, with other genes involved in necroptosis, apoptosis, and autophagy. This high network connectivity indicates that in the Severe group the biological functions and/or cellular processes related to innate immune response and inflammation are more activated than in the Mild group, shedding some light on the underlying transcriptomic mechanism, since the immune response to SARS-CoV-2 is characterized by neutrophil hyperactivation and high neutrophil counts^109^. Most of the patients of the Severe group included in this study presented high NLR as compared with the Mild group.

It is important to highlight that the young (subgroup A) and old patients (subgroup B) in the Severe group also presented differences in their transcriptomic profiles. Two modules—magenta and black—that are correlated with the subgroup A, contains many HH genes related to the innate immune response to viral infections, as well as other HH genes related to modulatory mechanisms acting on these responses. Moreover, in the black subnetwork the highest gene interconnectivity was found for subgroup A, thus indicating that immune modulatory functions are more activated in this subgroup when compared to subgroup B. These transcriptomic mechanisms could be related to the lower median hospital stay that has been observed for young patients (including our cohort) when compared with elderly patients^110^. In the elderly patients a less effective immune modulation of COVID-19, allied to immune senescence and, eventually, to comorbidities, would lead to a worse outcome^111^.

A total of 23 DEGs were found, all hyper-expressed in the Severe group. These genes are potential biomarkers for COVID-19 severity (13 are newly described). Remarkably, thirteen of these DEGs are involved in neutrophil activation, confirming the role of neutrophils in severe COVID-19^37^. Four DEGs are involved in the p53 signaling pathway, that has been associated with lymphopenia in severe COVID-19 patients^3^, and three with the Hippo pathway, recently associated with the antiviral host response in COVID-19^112^. Epidemiological studies show that age remains a relevant predictive factor for COVID-19 severity^111^, together with pre-existing conditions, such as obesity, DM and hypertension^113^. However, the molecular mechanisms determining COVID-19 severity are not yet well understood. Hence, there is a demand for biomarkers derived from comparative transcriptome analyses of mild and severe cases, combined with patients’ clinico-demographic and laboratory data. These biomarkers may well be useful for the better stratification of risk factors in COVID-19.

## Supplementary Information

Supplementary Information.

## Data Availability

The datasets generated during and/or analyzed during the current study are available from the corresponding author on reasonable request. All microarray raw data have been deposited in GEO public database (http://www.ncbi.nlm.nih.gov/geo, token ahwlmsiervcnfid), a MIAME compliant database, under accession number GSE193022. All data will be released with paper publication.
