# Supplementary material for: Blood leukocyte transcriptional modules and differentially expressed genes associated with disease severity and age in COVID-19 patients
Source: Sci Rep. 2023 Jan 17;13:898. doi: 10.1038/s41598-023-28227-6 (PMC9844197; doi:10.1038/s41598-023-28227-6)
Supplement: Supplementary file 1 — Supplementary Information. [file 41598_2023_28227_MOESM1_ESM.zip › Supplementary information_Bando et al_Rev/Supplementary information_Bando et al-Rev.docx]

*Supplementary Information*

**Blood Leukocyte Transcriptional Modules and Differentially Expressed Genes Associated with Disease Severity and Age in COVID-19 Patients**

Silvia Y. Bando ^1#^, Fernanda B. Bertonha ^1#^, Sandra E. Vieira ^1#^, Danielle B. L. de Oliveira ^2,3^, Vanessa N. Chalup ^3^, Edison L. Durigon ^3^, Patricia Palmeira ^1^, Ana Cristina P. Curi ^1^, Caroline S. Faria ^4^, Leila Antonangelo ^4^, Gerhard da P. Lauterbach ^5^, Fabiane A Regalio ^6^, Roberto M. Cesar Jr ^7^, and Carlos A. Moreira-Filho^1,^*

^1^ Department of Pediatrics, Faculdade de Medicina da Universidade de São Paulo, São Paulo, SP, 05403-900, Brazil.

^2^ Instituto Israelita de Ensino e Pesquisa Albert Einstein, Hospital Israelita Albert Einstein, São Paulo, SP, 01310-200, Brazil.

^3^ Department of Microbiology, Laboratory of Clinical and Molecular Virology, Institute of Biomedical Sciences, Universidade de São Paulo, São Paulo, SP, 05508-040, Brazil.

^4^ Laboratório de Investigação Médica (LIM03), Hospital das Clínicas, Faculdade de Medicina da Universidade de São Paulo, São Paulo, SP, 01246-903, Brazil

^5^ Department of Internal Medicine, Faculdade de Medicina da Universidade de São Paulo, São Paulo, SP, 01246-903, Brazil.

^6^ Divisão de Anestesia, Hospital das Clínicas da Faculdade de Medicina da Universidade de São Paulo, São Paulo, SP, 01246-903, Brazil.

^7^ Department of Computer Science, Instituto de Matemática e Estatística da Universidade de São Paulo, SP, 05508-040, Brazil.

Silvia Y. Bando, silvia.bando@fm.usp.br, https://orcid.org/0000-0003-3794-2440

Fernanda B. Bertonha, fernanda.bernardi@fm.usp.br, https://orcid.org/0000-0002-3675-1362

Sandra E. Vieira, sandra.vieira@fm.usp.br, https://orcid.org/0000-0001-5100-8713

Danielle B. L. de Oliveira, danibruna@usp.br, https://orcid.org/0000-0002-0534-0886

Vanessa N. Chalup, vmnchalup@usp.br, https://orcid.org/0000-0002-9433-0443

Edison L. Durigon, eldurigo@usp.br, https://orcid.org/0000-0003-4898-6553

Patricia Palmeira, patricia.palmeira@hc.fm.usp.br, https://orcid.org/0000-0002-6268-8141

Ana Cristina P. Curi, anacristina.pcuri@gmail.com, https://orcid.org/0000-0002-9480-7248

Caroline S. Faria, carolmbio@gmail.com, https://orcid.org/0000-0001-5272-2169

Leila Antonangelo, l.antonangelo@hc.fm.usp.br, https://orcid.org/0000-0002-8634-5100

Gerhard da P. Lauterbach, gerhardpaz@hotmail.com, https://orcid.org/0000-0002-1448-8153

Fabiane A Regalio, fabiane.aliotti@hc.fm.usp.br

Roberto M. Cesar Jr, rmcesar@usp.br, https://orcid.org/0000-0003-2701-4288

Carlos A. Moreira-Filho, cmoreira@usp.br, https://orcid.org/0000-0003-3433-4714

^*^ Correspondence: cmoreira@usp.br; Tel.: +55-11-97601-5087; +351-919-489-835

^#^ SYB, FBB and SEV contributed equally to this work

**Supplementary Methods**

Relative gene expression analysis. The relative expression of the differentially expressed genes (DEGs) was normalized with the endogenous reference gene *GUSB* for statistical analysis. This choice was made after evaluation of the standard deviation (SD) and the *p*-value (t-test) of some well-known housekeeping genes [Vandesompele et al., 2002; Chervoneva et al., 2010; Kozera et al., 2013; González-Bermudez et al., 2019] that were also present in our microarray data (**Table S3**). Those reference genes presenting a constant level of expression among the samples/groups were taken into consideration, until the final selection of *GUSB*, as it presented the lowest SD value an, also, the highest *p*-value.

**Supplementary Tables**

**Table S1**. Clinical and demographic data of the 121 patients

**Table S2**. Clinical and demographic data of the patients included in the transcriptomic analyses

**Table S3**. Selection of the endogenous reference gene most appropriate to our study. In bold, the gene of choice, GUSB

| **Gene** | **Average expression** | | **FC** | **SD** | ***p*-value** | **Reference** |
| --- | --- | --- | --- | --- | --- | --- |
|  | **Severe** | **Mild** | **Severe/Mild** |  |  |  |
| *ACTB* | 14.2725 | 14.3982 | -0.9 | 0.6 | 0.31 | Vandesompele et al., 2002 |
| *B2M* | 17.3072 | 17.0845 | 1.2 | 0.5 | 0.13 |  |
| *GAPDH* | 14.3112 | 13.6712 | 1.9 | 0.8 | 0.02 |  |
| *HPRT1* | 10.1691 | 9.9655 | 1.2 | 0.6 | 0.22 |  |
| *RPL13A* | 15.9702 | 16.0755 | -0.9 | 0.3 | 0.24 |  |
| *SDHA* | 9.5630 | 9.5325 | 1.0 | 0.5 | 0.44 |  |
| *UBC* | 16.9333 | 16.7601 | 1.2 | 3.5 | 0.06 |  |
| *YWHAZ* | 10.2121 | 10.7561 | -0.6 | 1.2 | 0.15 |  |
| *MRPS27* | 8.8132 | 8.3573 | 1.6 | 0.6 | 0.04 | Chervoneva et al., 2010 |
| *MRPS30* | 8.3182 | 7.9130 | 1.5 | 1.8 | 0.10 |  |
| *TFRC* | 9.3508 | 8.6852 | 1.9 | 0.7 | 0.01 |  |
| *LDHA* | 10.1654 | 9.4580 | 2.0 | 0.8 | 0.02 |  |
| *RPL32* | 16.1325 | 16.1897 | -0.9 | 5.6 | 0.36 | Kozera et al. 2013 |
| *PUM1* | 10.6453 | 10.6823 | -1.0 | 0.5 | 0.44 | Gonzáles-Bermúdez et al., 2019 |
| *TBP* | 8.3449 | 8.0702 | 1.3 | 0.5 | 0.08 |  |
| ***GUSB*** | **11.9832** | **12.0567** | **-0.9** | **0.4** | **0.33** |  |

**Table S4**. Reference values and range of the hemogram parameters used in this study

**References**

Greer JP, Rodgers GM, Glader B, Arber DA, Means, Jr RT, List AF, Appelbaum FR, Dispenzieri A, Fehniger TA. Wintrobe’s Clinical Hematology, 14th ed. Wolters Kluwer, Alphen aan den Rijn, Netherlands, 2018.

Naoum PC, Naoum FA. Hematologia Laboratorial: Eritrócitos, 2nd ed, Academia de Ciência e Tecnologia, São José do Rio Preto, Brazil, 2008.

Naoum FA, Naoum PC. Hematologia laboratorial: Leucócitos. 1st ed, Academia de Ciência e Tecnologia, São José do Rio Preto, Brazil, 2006.

Gonçalves J, Souza NMA, Hermes EM, Jesus CSC, Jordão MM. Perfil hematológico dos neonatos atendidos no Hospital Universitário da Universidade Federal de Santa Catarina. Rev Bras Hematol Hemoter. 2010; 32(3):2019-24.

Manual de Exames, Laboratório Fleury, São Paulo, Brazil, 2021

**Table S5**. Comparative analyses of the hemogram-derived ratios between Severe and Mild groups.

| **Hemogram-derived ratio** | **Severe (mean, STD)** | | **Mild (mean, STD)** | | | | | ***p-*value** | | |
| --- | --- | --- | --- | --- | --- | --- | --- | --- | --- | --- |
|  | A (10-40yrs) | B (41-80yrs) | | C (10-40yrs) | D (41-80yrs) | |  | | |  |
| **NLR** | **4.99 (5.64)** | | **1.68 (1.86)** | | | | | **< 0.0001** | | |
| Ratio value (mean, STD) | 3.35 (2.01) | 5.95 (6.67) | | 1.38 (0.82) | | 2.42 (2.88) | | |  | |
| *p*-value | NS | | 0.009 | | | | |  | | |
| **NPR** | **2.04 (1.14)** | | **1.25 (0.62)** | | | | | **0.0002** | | |
| Ratio value (mean, STD) | 2.00 (1.20) | 2.20 (1.10) | | 1.20 (0.53) | | 1.50 (0.71) | | |  | |
| *p*-value | NS | | NS | | | | |  | | |
| **PLR** | **0.21 (0.33)** | | **0.09 (0.07)** | | | | | **< 0.0001** | | |
| Ratio value (mean, STD) | 0.11 (0.03) | 0.27 (0.40) | | 0.08 (0.05) | | 0.11 (0.09) | | |  | |
| *p*-value | 0.0055 | | 0.0417 | | | | |  | | |
| **SII** | **16.86 (31.00)** | | **4.89 (5.75)** | | | | | **< 0.0001** | | |
| Ratio value (mean, STD) | 7.10 (2.90) | 22.00 (38.00) | | 4.10 (3.80) | | 6.90 (8.20) | | |  | |
| *p*-value | 0.0191 | | 0.0166 | | | | |  | | |
| NLR: neutrophil-lymphocyte ratio; NPR: neutrophil-to-platelet ratio; PLR: Platelet-to-lymphocyte ratio; SII: systemic immune-inflammation index; STD: Standard Deviation; NS: Not significant; yrs: years. All statistical analyses were performed by Mann-Whitney test. | | | | | | | | | | |

**Table S6.** Enrichment analyses for the yellow module. The terms that appear in the histogram (Figure 6) are highlighted in grey. In bold, HH genes

**Table S7.** Enrichment analyses for the magenta module. Terms that appear in the histogram (Figure 7) are highlighted in grey. In bold, HH genes

**Table S8.** Enrichment analyses for the black module. Terms that appear in the histogram (Figure 8) are highlighted in grey. In bold, HH genes

**Table S9.** Enrichment analyses for DEGs obtained from the Severe vs Mild group comparison. Terms in bold contain DEGs selected as potential biomarkers. The terms highlighted in grey appear in the histogram (Figure 9)

**Table S10.** List of the genes used for the construction of the modules’ subnetworks

**Supplementary Figures**

**
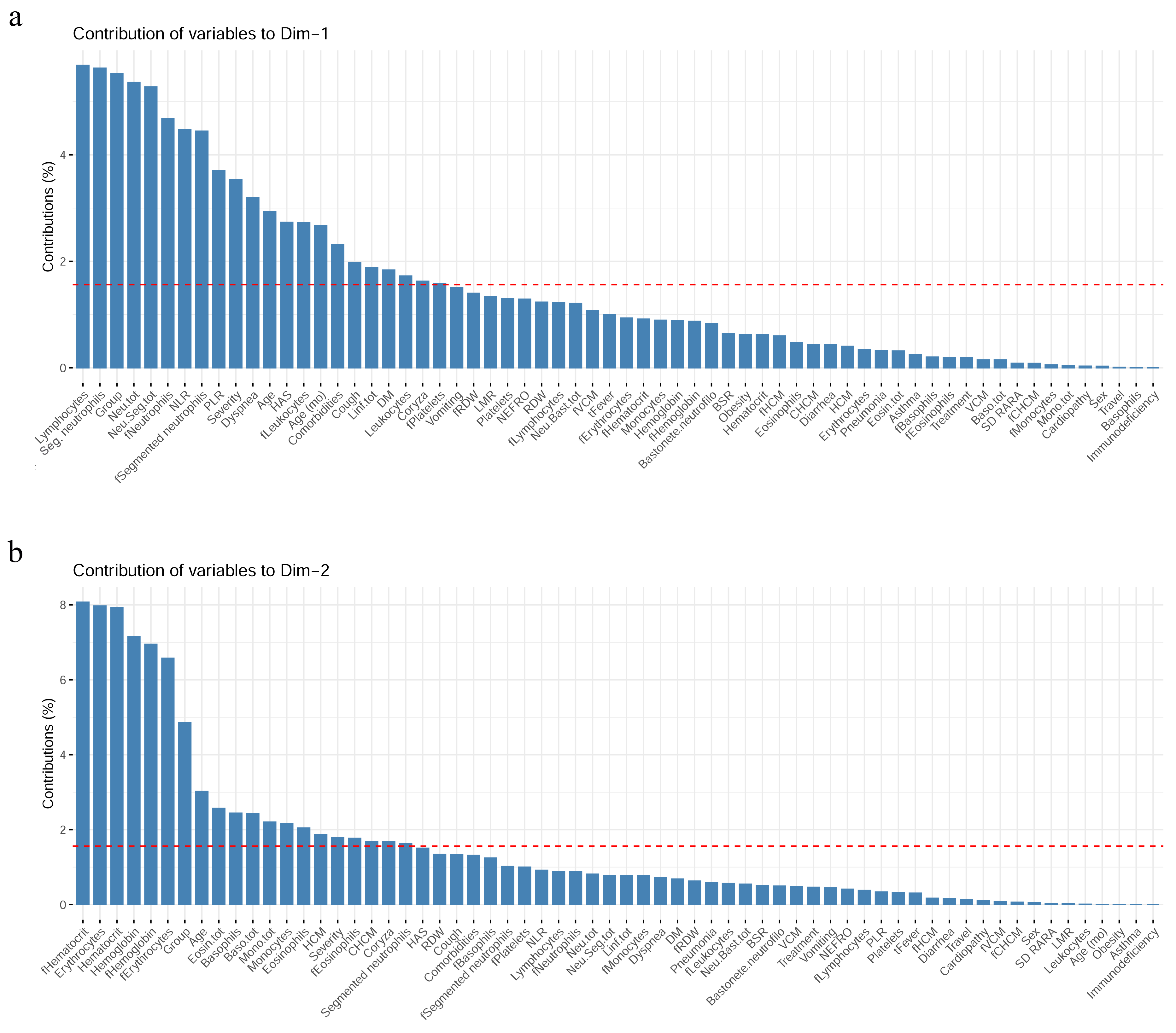
**

**Figure S1.** Factor Analysis of Mixed Data. Contribution of grouping variables to the first (**a**) and second (**b**) dimensions.

**Figure S2.** Boxplot for the gene expression raw data matrices of the 23 samples included in the WGCNA (**a**) or DEG analyses (**b**).


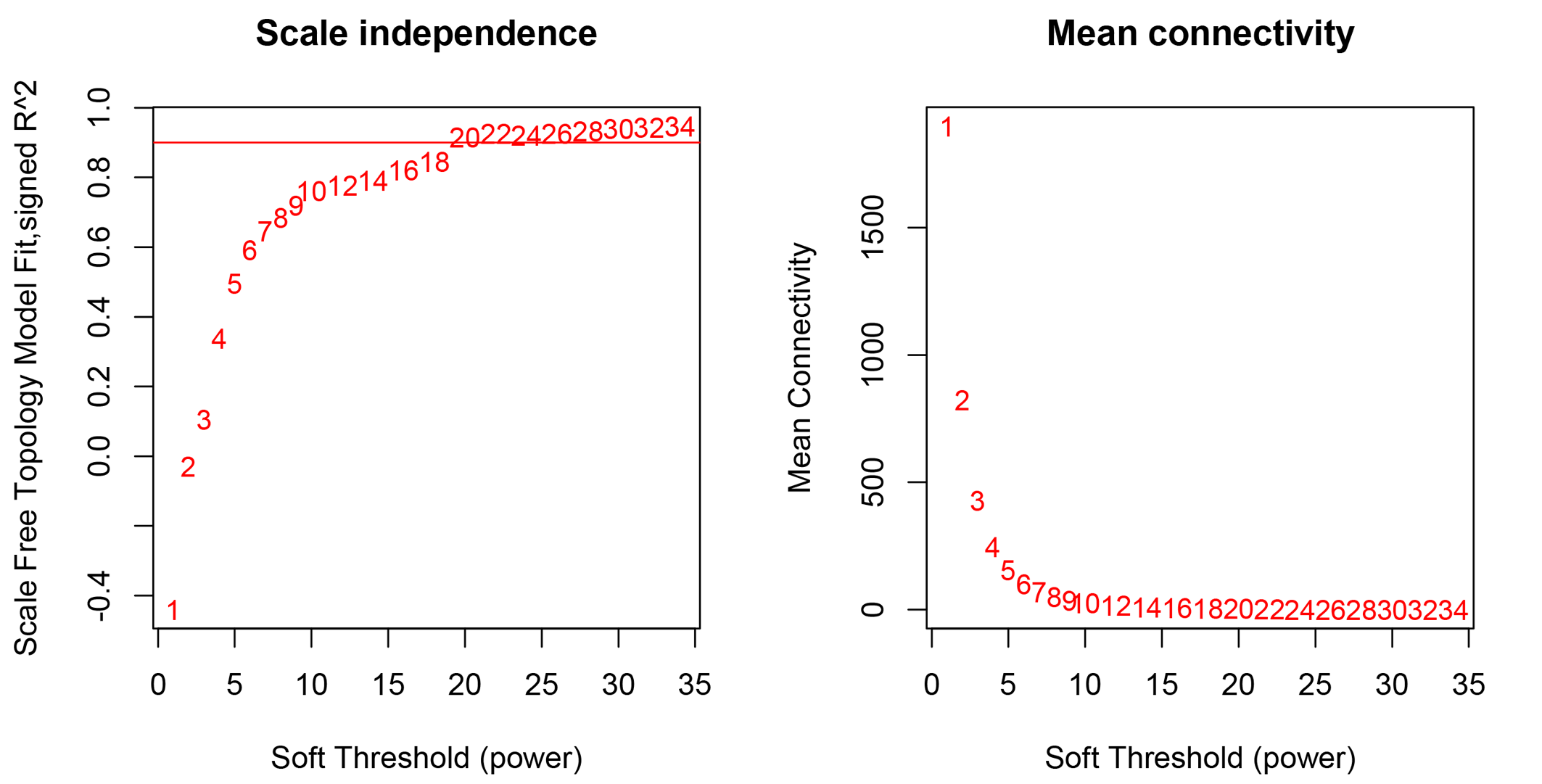


**Figure S3.** Selection of the soft-thresholding power (β). The dataset was fit to a scale-free model of proposed values for β ranging from 1 to 34 (numbers inside the plots). Approximate scale-free topology is attained around soft-thresholding power of 20, which reflects the inflection point where model fit begins to decrease with power increasing (left panel). The plot of the mean connectivity along with the soft-thresholding power (right panel). The red line indicates the scale-free topology R^2^ fit index cut-off of 0.9160.


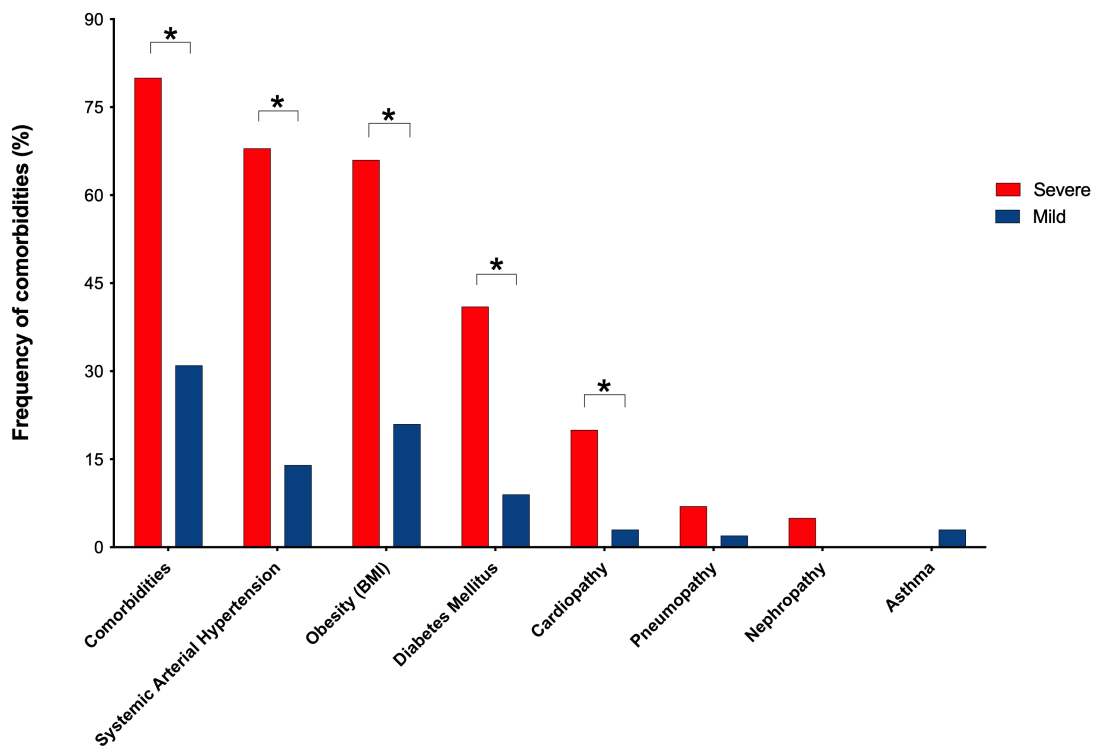


**Figure S4.** Frequency of comorbidities in COVID-19 patients. Frequency of comorbidities among the patients (n = 121) with confirmed SARS-CoV-2 infection. Severe and mild phenotypes are depicted by red and blue bars, respectively. Differences were tested using one-tailed Mann-Whitney t-test (**p* < 0.01). *Stands for comorbidities significantly correlated with COVID-19 patients with a severe outcome. Pneumopathy includes chronic lung diseases: bronchiectasis, bronchopulmonary dysplasia, chronic obstructive pulmonary disease, (including emphysema and chronic bronchitis), interstitial lung disease, pulmonary embolism, and pulmonary hypertension. Cardiopathy includes chronic heart conditions such as heart failure, coronary artery disease, cardiomyopathies, and arterial hypertension. Nephropathy includes chronic kidney disease.


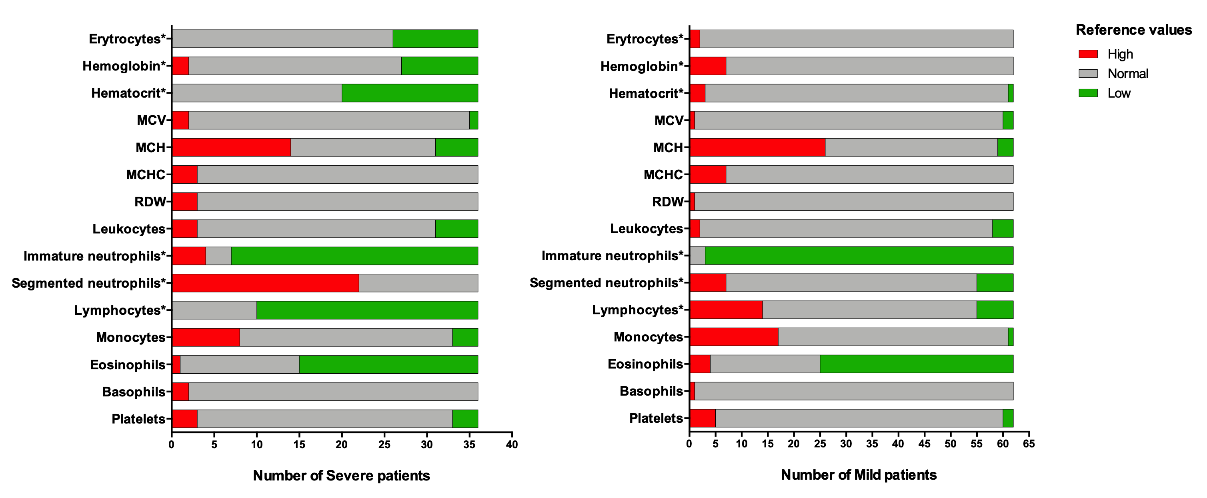


**Figure S5.** Hematological findings in COVID-19 patients. Hematological findings for both severe (left panel) and mild (right panel) phenotypes. Colored bars indicate the number of patients presenting high blood count values (in red), low blood count values (in green), and normal blood count reference values (in grey). *Significant correlation with severity was found for high values of immature and segmented neutrophils, and low levels of erythrocytes, hemoglobin, hematocrit, and lymphocytes (*p* < 0.005). MCV: Mean Corpuscular Volume, MCH: Mean Corpuscular Hemoglobin, MCHC: Mean Corpuscular Hemoglobin Concentration, RDW: Red cell Distribution Width.


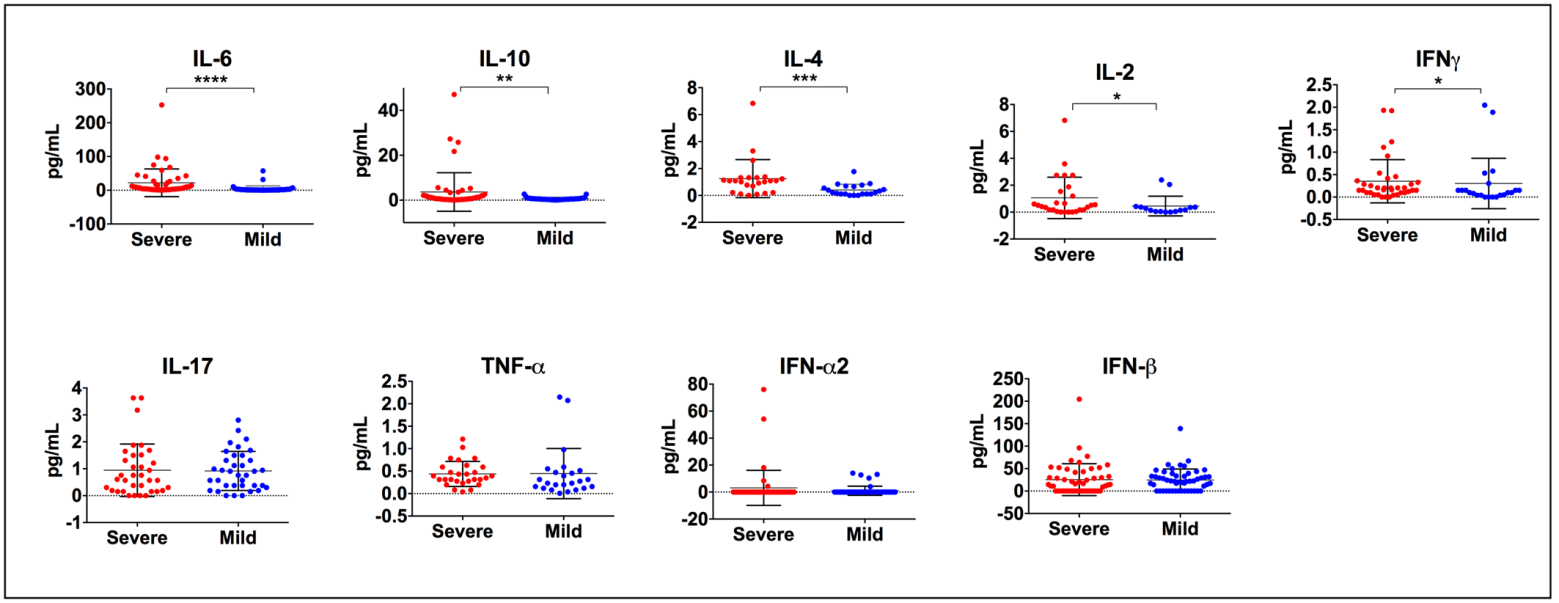


**Figure S6.** Plasmatic cytokine levels. The dot plots show the comparisons of plasmatic cytokine (IL-2, IL-4, IL-6, IL-10, IL-17, TNF-α, IFN-α2, and IFN-β) concentrations (pg/mL) between severe and mild cases. The data are presented as the mean with SD. Differences were tested using one-tailed Mann-Whitney t-test (**** *p* < 0.00001; *** *p* < 0.0001; ** *p* < 0.001; * *p* < 0.01).

**Figure S7.** Module-trait relationships (complete report). WGCNA module significance (MS) correlations with clinical, laboratorial, and/or demographic traits. In the rows, module eigengenes (MEs) named by their module colors, together with the number of genes in each module. In the columns, the traits of interest. Numbers inside each colored box are the correlation coefficients between the module and the specific trait, with *p*-value in parentheses. The more intense the box color, the more negatively (green) or positively (red) is the module correlation with the trait (MS value, as indicated by color bar). Msevere, male in the Severe group; Mmild, male in the Mild group; DM_Severe, severe patients with DM; ObeSevere, obese severe patients; SAH, Systemic Arterial Hypertension; Anti-SAH, anti-hypertensive drugs; H-SN, high level of segmented neutrophils; N-SN, normal level of segmented neutrophils; H-Lymphs, high level of lymphocytes; L-Lymphs, low level of lymphocytes; N-Lymphs, normal level of lymphocytes. Module-trait association analysis was accomplished using the WGCNA package (version 1.69–81; <https://horvath.genetics.ucla.edu/html/CoexpressionNetwork/Rpackages/WGCNA/>) in R version 3.6.2 environment.

**
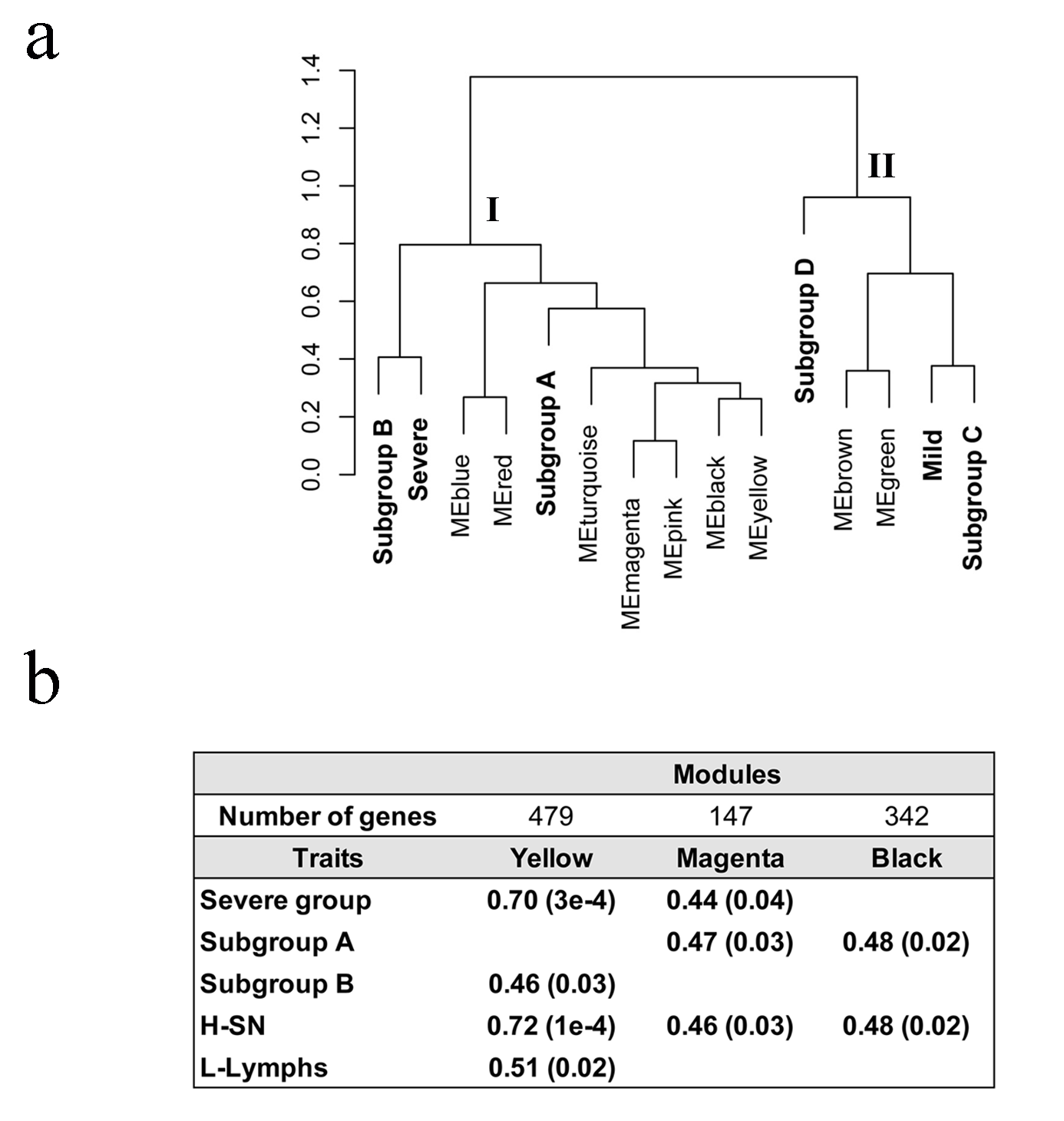
**

**Figure S8.** Module-trait relationships. Eigengene dendrogram shows the correlation between modules and traits (severity and age) (**a**). Significant module-trait positive correlations with severity groups, age subgroups, and neutrophil and lymphocyte levels are depicted in (**b**). The traits of interest appear in the rows and the modules appear in the columns. The numbers stand for the correlation coefficients between the module and a specific trait, with the *p*-values between parentheses. Only the positive module-trait correlations are shown (*p* < 0.05). H-SN: high level of segmented neutrophils, L-Lymphs: low level of lymphocytes.


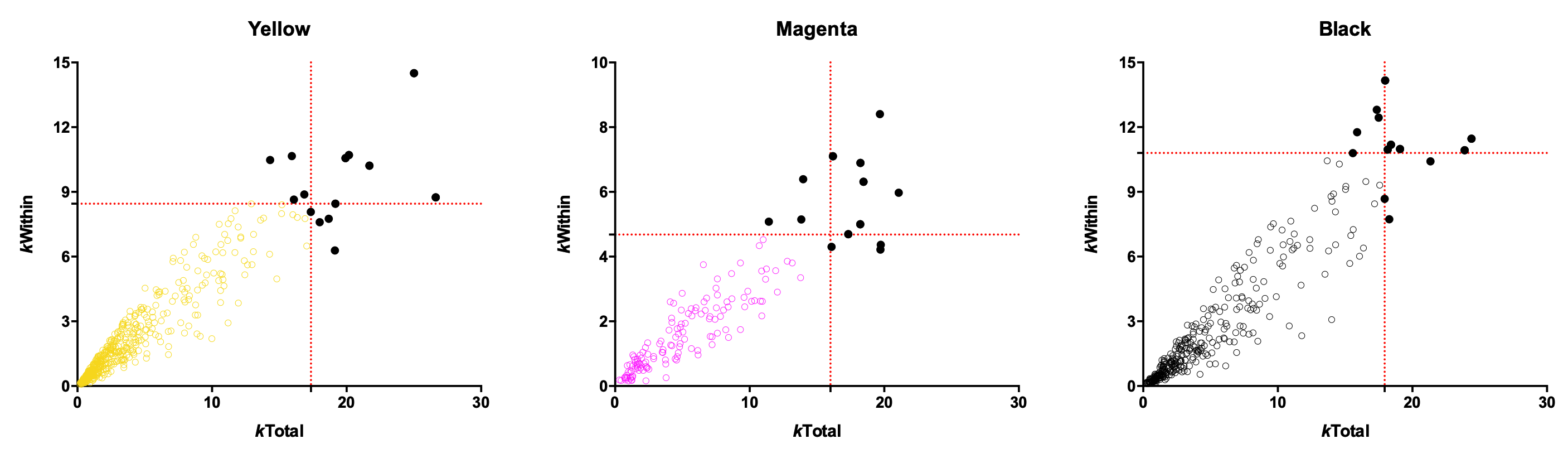


**Figure S9.** Intramodular node categorization. *k*Total *vs.* *k*Within plots for the three modules (yellow, magenta, and black) significantly and positively correlated with specific traits. High hierarchy (HH) genes are indicated by black dots. Red dotted lines on both axes show the cut-off values adopted for HH genes selection.


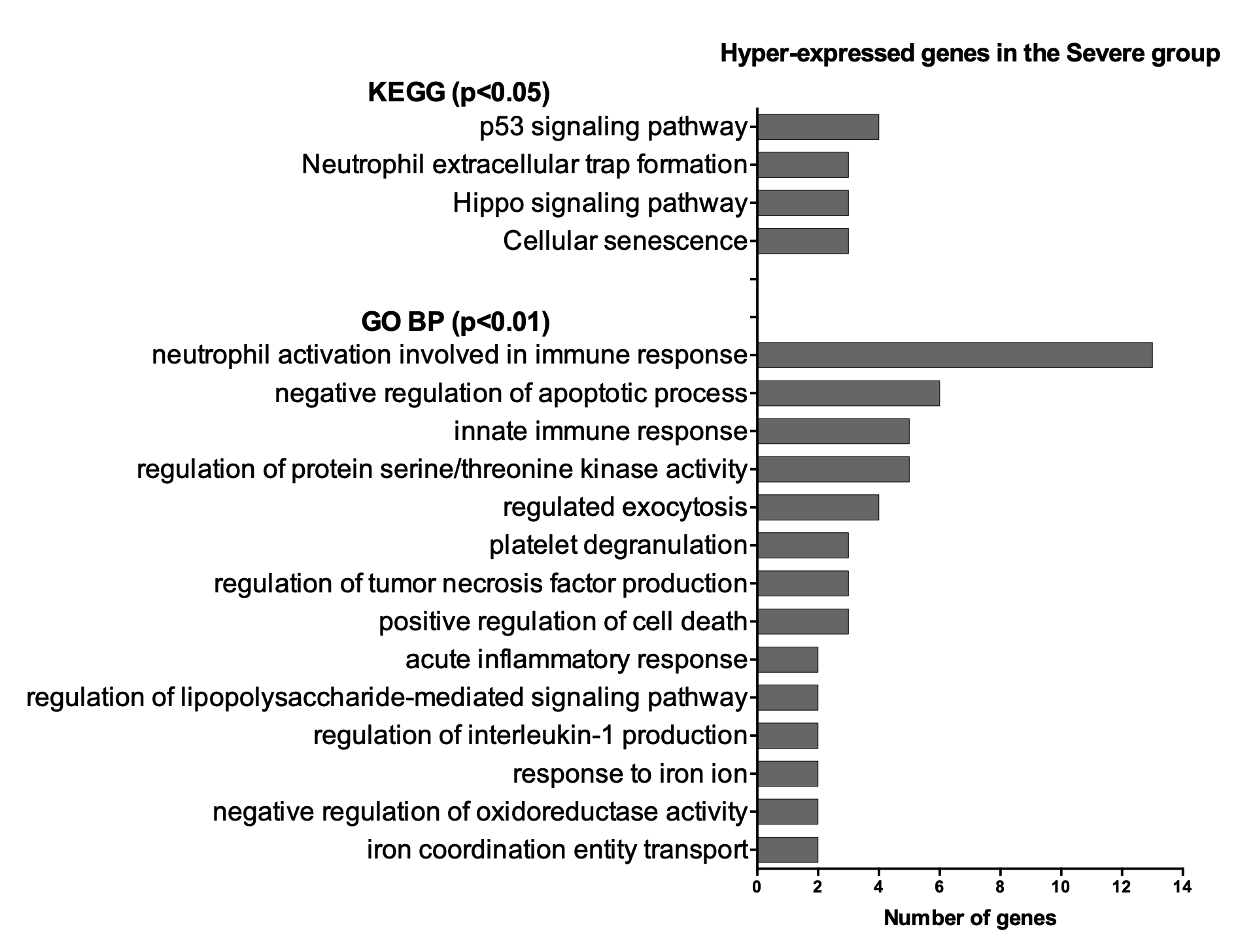


**Figure S10.** Histogram of enriched GO BP and KEGG pathways terms for the DEGs and hyper-expressed genes in the Severe group. The terms with *p* < 0.05 were considered significant.


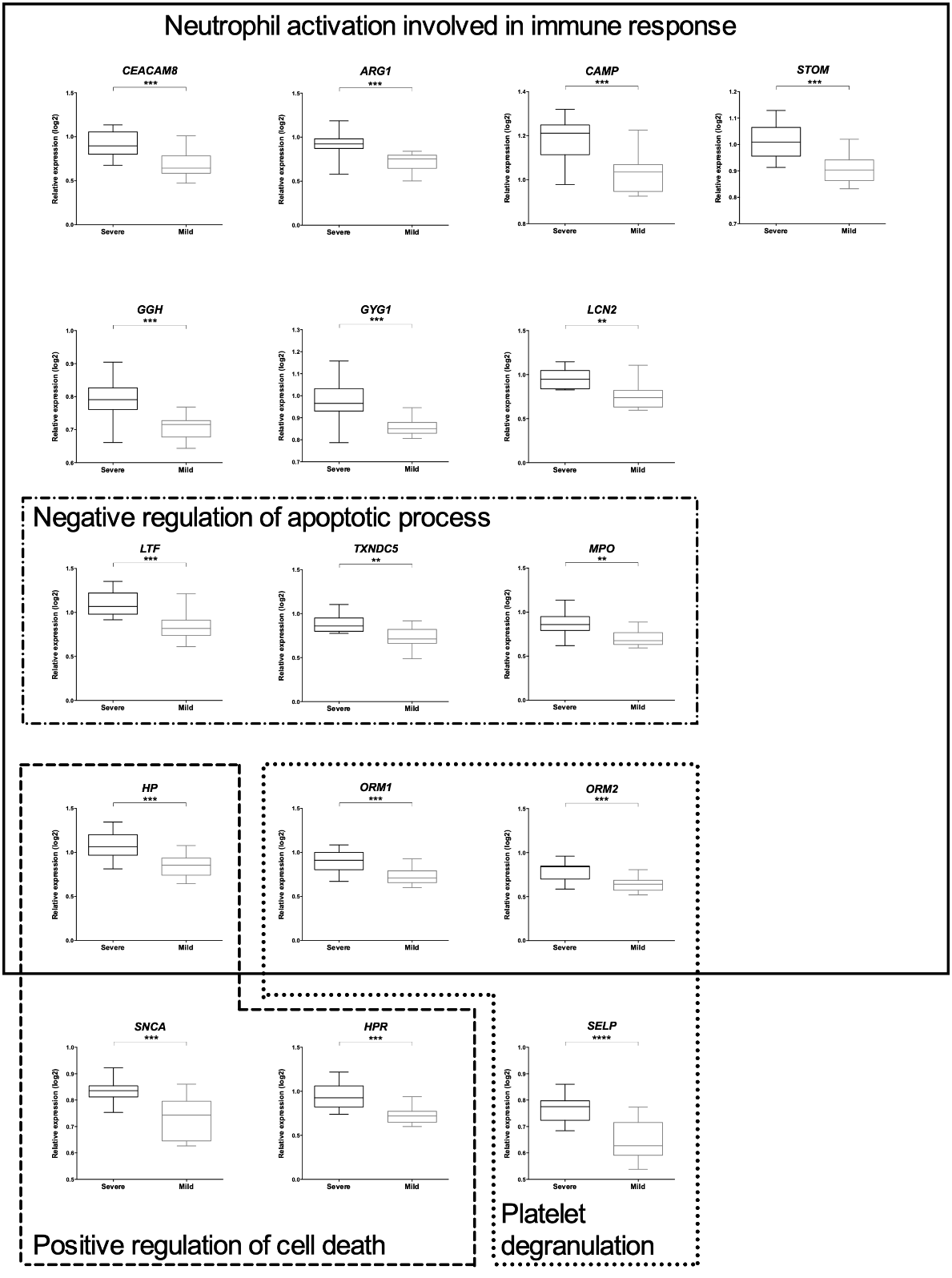


**Figure S11.** Relative expression of the DEGs related to neutrophil activation, negative regulation of apoptotic process, positive regulation of cell death, and platelet degranulation. The boxplots show the relative expression of genes involved in innate immune response. The relative expression of the differentially expressed genes was normalized with the endogenous reference gene *GUSB* for statis-tical analysis. Differences were tested using unpaired one-tailed t-test (**** *p* < 0.00001; *** *p* < 0.0001; ** *p* < 0.001)


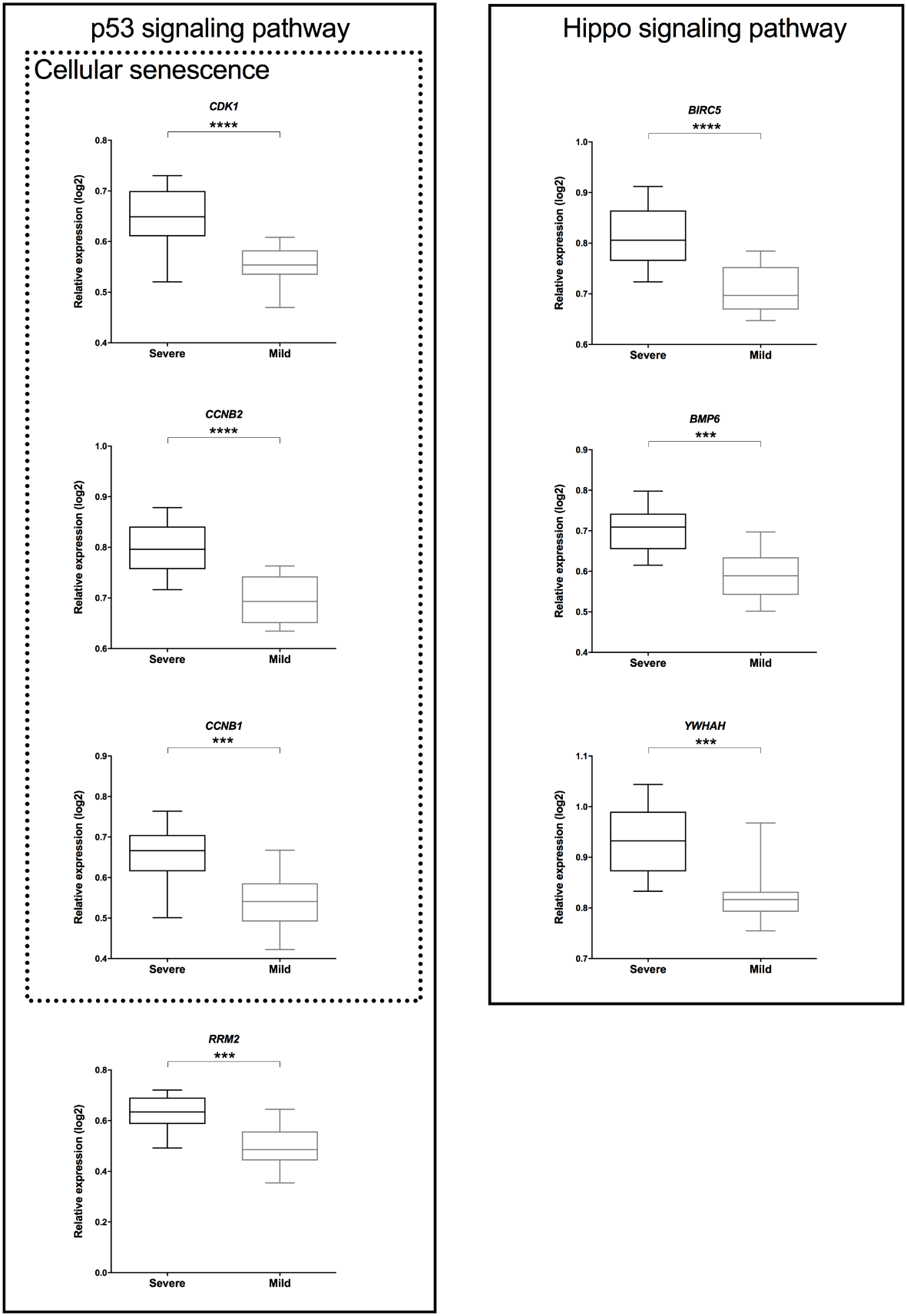


**Figure S12.** Relative expression of the DEGs related to p53 and Hippo signaling pathways. Boxplots show the relative expression of genes related to cellular senescence or cellular senescence-related signaling pathways. The relative expression of the differentially expressed genes was normalized with the endogenous reference gene *GUSB* for statistical analysis. Differences were tested using unpaired one-tailed t-test (**** *p* < 0.00001; *** *p* < 0.0001)
